# Rapid Blood Clot Removal via Remote Delamination and Magnetization of Clot Debris

**DOI:** 10.1002/advs.202415305

**Published:** 2025-03-08

**Authors:** Qinglong Wang, Ben Wang, Kai Fung Chan, Xin Song, Qianqian Wang, Fengtong Ji, Lin Su, Bonaventure Yiu Ming Ip, Ho Ko, Philip Wai Yan Chiu, Thomas Wai Hong Leung, Li Zhang

**Affiliations:** ^1^ College of Chemistry and Environmental Engineering Shenzhen University Shenzhen China; ^2^ Department of Mechanical and Automation Engineering The Chinese University of Hong Kong (CUHK) Shatin, N.T. Hong Kong China; ^3^ Chow Yuk Ho Technology Center for Innovative Medicine CUHK Shatin N.T. Hong Kong China; ^4^ Multi‐Scale Medical Robotics Center Hong Kong Science Park Shatin N.T. Hong Kong SAR China; ^5^ Jiangsu Key Laboratory for Design and Manufacture of Micro‐Nano Biomedical Instruments School of Mechanical Engineering Southeast University Nanjing China; ^6^ Division of Neurology Department of Medicine and Therapeutics CUHK Shatin N.T. Hong Kong China; ^7^ Department of Surgery CUHK Shatin N.T. Hong Kong China; ^8^ CUHK T Stone Robotics Institute CUHK Shatin N.T. Hong Kong China

**Keywords:** catheterization, microswarm, rapid thrombus removal, remote delamination

## Abstract

Micro/nano‐scale robotic devices are emerging as a cutting‐edge approach for precision intravascular therapies, offering the potential for highly targeted drug delivery. While employing micro/nanorobotics for stroke treatment is a promising strategy due to its ability to localize therapy and minimize drug dosage, current methods require prolonged treatment durations, increasing the risk of nerve tissue necrosis from extended hypoxia. Here a programmable colloidal microswarm capable of rapidly detaching blood clots from the vessel wall is developed, enabling swift recanalization without the need for complete clot degradation. More importantly, the detached clot debris, despite their random shapes, functions as magnetic “debris‐robots” and can be efficiently propelled through helical swimming within flowing vessels, followed by retrieval using catheter suction. The entire process—including catheter delivery, controlled locomotion, clot detachment, and retrieval—can be completed in approximately half an hour, significantly saving time compared to the critical “Golden 6 hours” window for stroke treatment. This retrieval procedure greatly minimizes nanoparticle exposure in the bloodstream and lowers the risk of secondary clotting in distal vessels, marking a significant advancement in robotic‐assisted thrombolysis.

## Graphical abstract

A programmable colloidal microswarm is developed to quickly detach blood clots and enable rapid recanalization, with the detached debris functioning as a magnetic “debris‐robot” that can be efficiently propelled and retrieved via catheter suction, which could significantly save time compared to the critical “Golden 6 hours” window for ischemic stroke treatment.

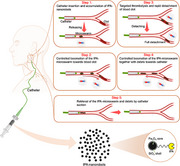

## 1 Introduction

Vascular occlusions caused by blood clots restrict normal blood flow and hinder nutrient and oxygen supply, resulting in many life‐threatening diseases such as ischemic stroke, pulmonary embolism, and myocardial infarction.^[^ ^1^ ^]^ Large‐scale trials in acute ischemic stroke treatment indicate that the odds of being alive significantly increased within 6 h (The “Golden 6 hours”). Efficient recanalization is crucial for saving lives and avoiding permanent brain damage. Realizing fast recanalization is vital for occlusion treatment; otherwise, serious sequelae and even death may occur. Current clinical treatments for thrombosis include mechanical thrombectomy, which uses catheters to physically remove blood clots, and thrombolytic therapy with drugs like tissue plasminogen activator (tPA) to dissolve clots. However, thrombectomy shows its limitation in reduced effectiveness in navigating smaller or highly branched vessels, as well as the risk of vascular rupture due to the thin and fragile walls of these vessels. Thrombolysis using tPA shows its limitation in short half‐life, rapid clearance, non‐targeted circulation, and the risk of symptomatic intracranial hemorrhages (SIH), which are associated with high mortality and poor prognosis.^[^ ^2^ ^]^ To tackle those issues, novel delivery agents have been developed for localized thrombolysis to accelerate the recanalization and minimize the drug dose.^[^ ^1^, ^3^ ^]^

More recently, micro/nanorobotic technologies have been developed to remove blood clots by photothermal ablation, chemical degradation, or mechanical grinding.^[^ ^4^ ^]^ Those tiny machines achieve controllable locomotion by energy conversion, possessing exceptional targeting ability, enhanced accessibility, and high flexibility,^[^ ^5^ ^]^ which has been widely applied for drug delivery,^[^ ^6^ ^]^ microsurgery,^[^ ^7^ ^]^ water purification,^[^ ^8^ ^]^ bacteria killing,^[^ ^9^ ^]^ sensing,^[^ ^10^ ^]^ cancer therapy,^[^ ^11^ ^]^ etc. For instance, Zhao et al. reported using nanorobots to accelerate the thrombolysis by elevating drug transport through hydrodynamic convection, and the thrombolysis rate was improved up to 2‐fold compared with pure tPA.^[^ ^4k^ ^]^ Besides, a platelet‐derived nanomotor was developed and triggered by NIR irradiation to achieve desirable sequential drug release of thrombolytic urokinase and anticoagulant heparin for targeted thrombolysis.^[^ ^4h^ ^]^ Moreover, Wang et al. presented a reconfigurable magnetic microswarm that could enhance the thrombolysis effect by fluid convection, and the degradation rate was enhanced 2.5‐fold compared with that without the microswarm.^[^ ^12^ ^]^ Micro/nanorobots are able to accelerate the thrombolysis process and reduce the drug dose attributed to the localized therapy. As micro/nanorobots may contain non‐biodegradable components, the safety issue becomes one of the primary challenges for intravascular therapeutics. The micro/nanorobots circulating in the vascular system may cause blood vessel blockage, potential immune response, carcinogenic effects, and other unexpected side effects. More importantly, prolonged treatment duration using current micro/nanorobotics for vascular recanalization poses a threat of compromised outcomes due to nerve tissue necrosis caused by sustained hypoxia.

**Figure 1:**
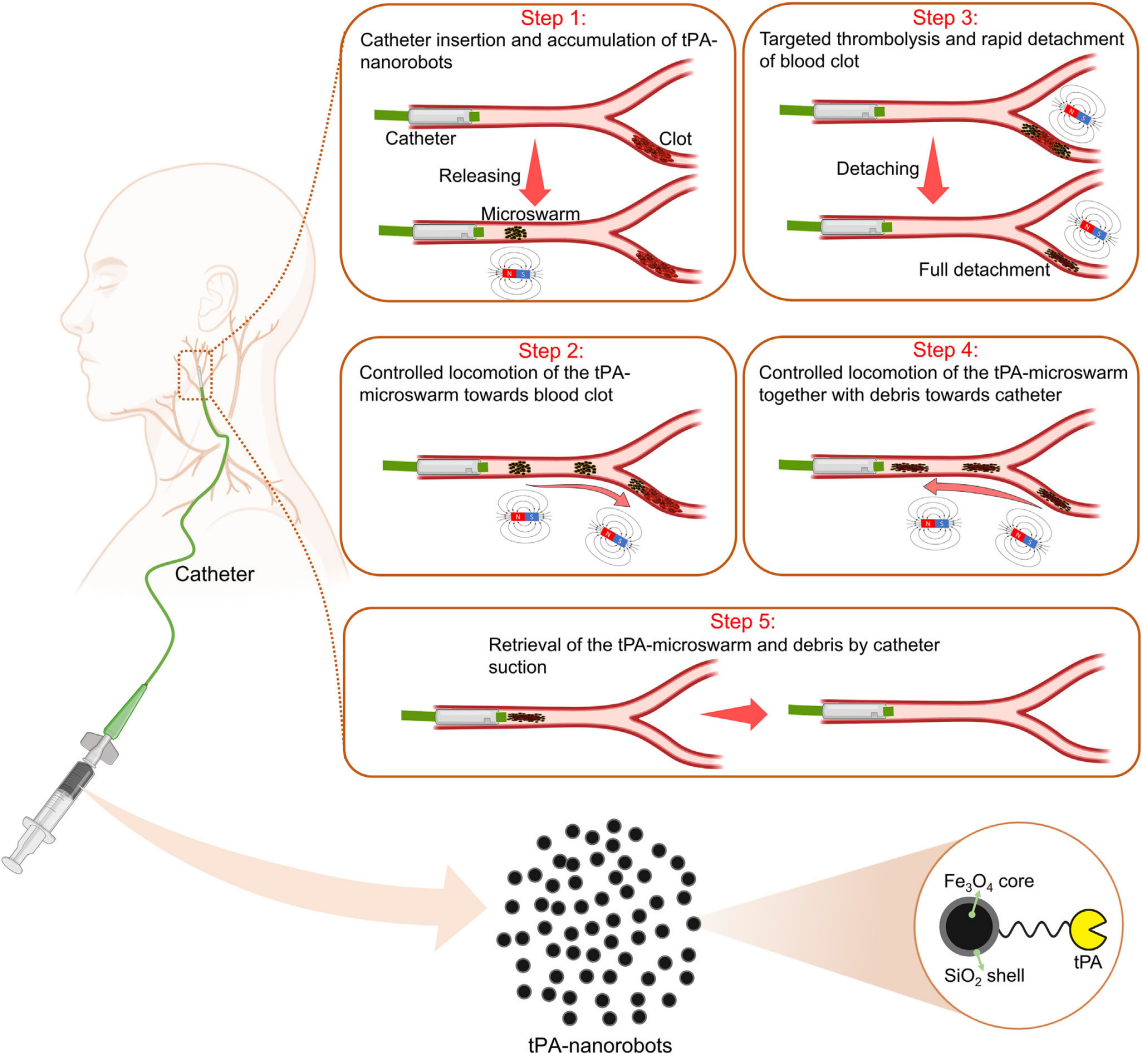
The schematic shows the whole process for rapid recanalization and retrieval of the blood clot using the tPA‐microswarm and a catheter. Step 1, catheter insertion and accumulation of tPA‐microswarm; Step 2, controllable locomotion of the tPA‐microswarm toward the blood clot; Step 3: targeted thrombolysis and detachment of the blood clot; Step 4: controllable locomotion of the tPA‐microswarm together with debris toward the catheter; Step 5: retrieval of the tPA‐microswarm and debris by catheter suction. Partial of the schematic was created using BioRender.com.

To address the challenges, we design a functionalized magnetic colloidal microswarm, with tPA anchored on the highly porous surface of the colloidal particles (tPA‐microswarm). The microswarm can preferentially degrade the blood clot along the vessel wall and further rapidly detach the blood clot by applying a designed magnetic field. Our approach offers a distinct advantage over prior methods by enabling rapid recanalization without requiring complete clot degradation (**Figure** **1**). Quantitative results demonstrate effective recanalization can be accomplished within just 20 min when addressing a blood clot measuring 7 mm in length within the 1.5‐mm vessel. More importantly, from the integration of the magnetic navigation setup with the clinical catheter, the colloidal microswarm can be retrieved, along with the detached blood clot debris, using a catheter‐assisted magnetic swimming process to prevent prolonged exposure to nanoparticles in the bloodstream and secondary occlusion, thus reducing potential side effects. The entire process, encompassing catheter delivery, controllable locomotion, rapid detachment, and retrieval, can be accomplished in just 32 min. Compared to catheter‐based thrombectomy, our magnetic microswarm can navigate into these hard‐to‐reach regions, enabling rapid and effective clot disruption while minimizing the risks of vessel damage or distal embolization. Additionally, the non‐invasive, remotely controlled nature of our system minimizes the risk of vessel damage. The proposed colloidal microswarm will offer a new strategy for minimally invasive therapy in endovascular environments with substantially reduced side effects.

## 2 Results and Discussion

### 2.1 Material Characterizations

**Figure 2:**
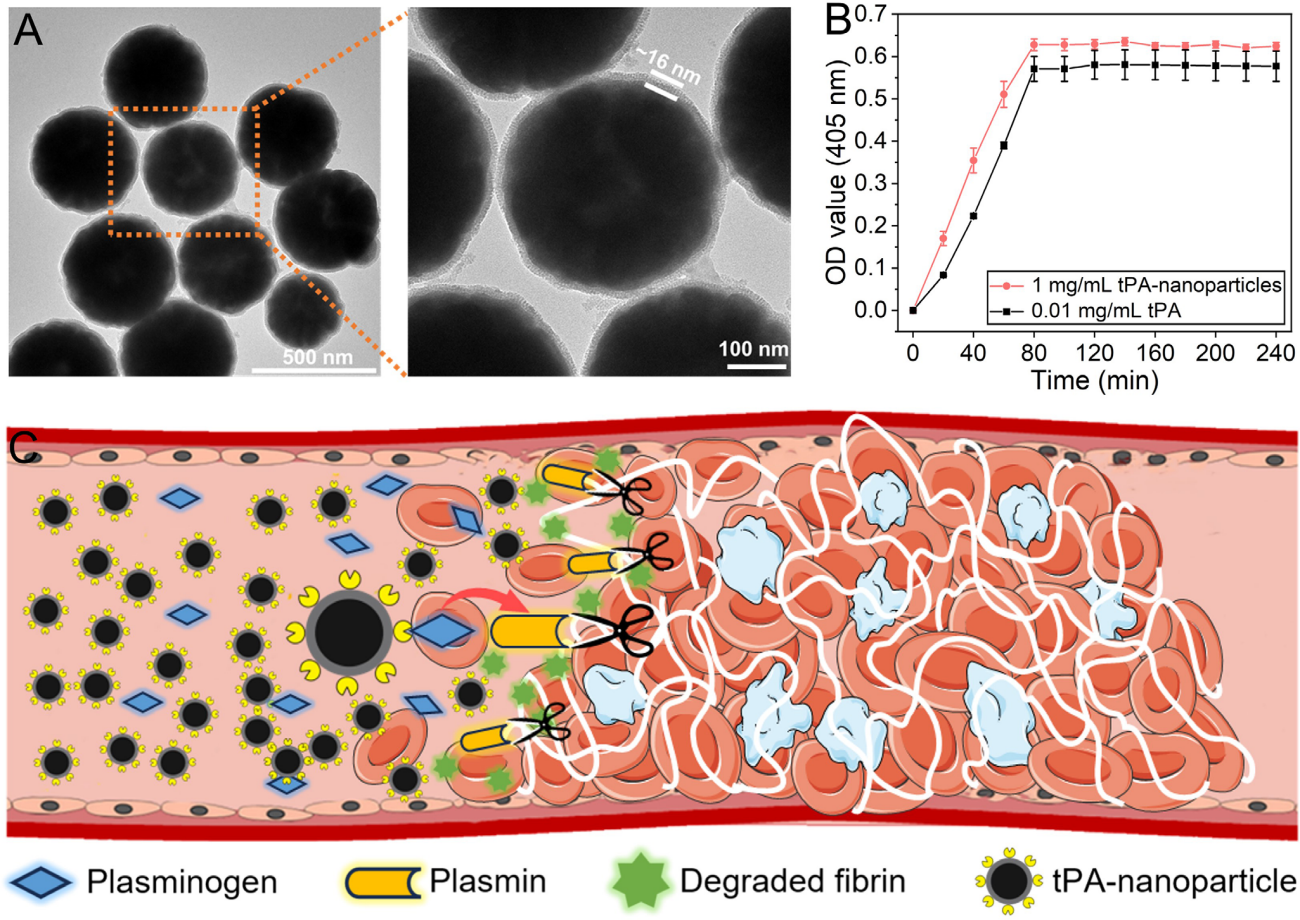
Material characterizations and mechanism of thrombolysis. A) Transmission electron microscope (TEM) image of Fe_3_O_4_@SiO_2_‐tPA NNPs. B) The curves show the OD value of tPA NNPs and pure tPA at 405 nm over the incubation time. C) The scheme illustrates the mechanism of the degradation process of the blood clot using tPA‐modified NNPs. The error bars donate the standard deviation (SD) from three experiments.

As shown in the TEM image (**Figure** **2**A), Fe_3_O_4_ nanoparticles (NNPs) exhibit uniform size distribution, and the diameter of NNPs is ≈300–400 nm. A SiO_2_ shell with a thickness of ≈16 nm is clearly observed on the surface of the Fe_3_O_4_ sphere. The mesoporous structure of the SiO_2_ shell allows more thrombolytic drugs (tPA) to bond on the surface. By comparison, no obvious SiO_2_ shell can be found in the TEM image of pure Fe_3_O_4_ NNPs (Figure S1, Supporting Information). To further verify the composition of the prepared samples, XRD measurements were conducted for both Fe_3_O_4_@SiO_2_ and Fe_3_O_4_ NNPs (Figure S2, Supporting Information). The XRD pattern of Fe_3_O_4_ NNPs was completely matched with the standard spectrum of PDF 88–0866, indicating the successful synthesis of magnetite. Moreover, Fe_3_O_4_@SiO_2_ NNPs showed a very similar pattern compared with Fe_3_O_4_ NNPs, suggesting that SiO_2_ coating exerted an insignificant influence on the crystal structure of magnetite. Moreover, the results of zeta potential and dynamic light scattering (DLS) for various NNPs were offered (Figure S3, Supporting Information). The zeta potential of prepared Fe_3_O_4_ NNPs was ≈3.5 mV, and the value changed to −19.4 mV after SiO_2_ coating due to the substantial carboxyl groups of Fe_3_O_4_@SiO_2_ NNPs. After amination and tPA modification, the zeta potential of Fe_3_O_4_@SiO_2_‐tPA NNPs became positive (≈6.5 mV). The DLS test was further conducted to verify the particle diameter in the solution. The size distribution of Fe_3_O_4_ NNPs was mainly located in the range of ≈250 to 530 nm, which was consistent with the TEM result. After SiO_2_ and tPA coating, the size of Fe_3_O_4_@SiO_2_, and Fe_3_O_4_@SiO_2_‐tPA NNPs slightly improved with average diameters of ≈400 and 460 nm, respectively. To further confirm the successful SiO_2_ coating, the element mapping of Fe_3_O_4_ and Fe_3_O_4_@SiO_2_ NNPs was evaluated in Figure S4 (Supporting Information). The SEM image showed the relatively uniform size distribution of Fe_3_O_4_ and Fe_3_O_4_@SiO_2_ NNPs. By comparison, much stronger element signals of Si can be obvious in the EDS image of Fe_3_O_4_@SiO_2_ NNPs. And Si element of Fe_3_O_4_@SiO_2_ NNPs counted ≈0.6 wt.% of the total element, whereas no significant Si element was detected in the spectrum of Fe_3_O_4_ NNPs (Figure S4C,D, Supporting Information). The results firmly verified the SiO_2_ coating on the synthesized Fe_3_O_4_@SiO_2_ NNPs. The enzyme activity of tPA‐NNPs was verified by a micro‐plate reader, and the curves exhibit the change of OD value over time (Figure 2B). The OD value of tPA‐NNPs (1 mg mL^−1^) drastically increased within 80 min, then reached the highest value of 0.6. By comparison, the OD value of pure tPA (0.01 mg mL^−1^) was slightly lower than that of tPA‐NNPs, which proved that tPA‐NNPs possessed a high enzyme activity. Contrary to previous methods requiring complete clot removal and extended treatment time, here, we propose a novel strategy to restore the blood flow via the fast detachment of the blood clot using a tPA‐microswarm. Figure 2C shows the mechanism of thrombolysis using tPA‐modified nanoparticles. tPA facilitates the conversion of plasminogen to plasmin, enabling the enzymatic breakdown of fibrin in blood clots, thus restoring vascular patency. Therefore, tPA plays a critical role in clot dissolution, dominantly impacting recanalization efficacy, meanwhile, our results confirm the successful modification of tPA on NNPs without the significant decline in enzyme activity.

### 2.2 Fast Detachment of the Blood Clot

**Figure 3:**
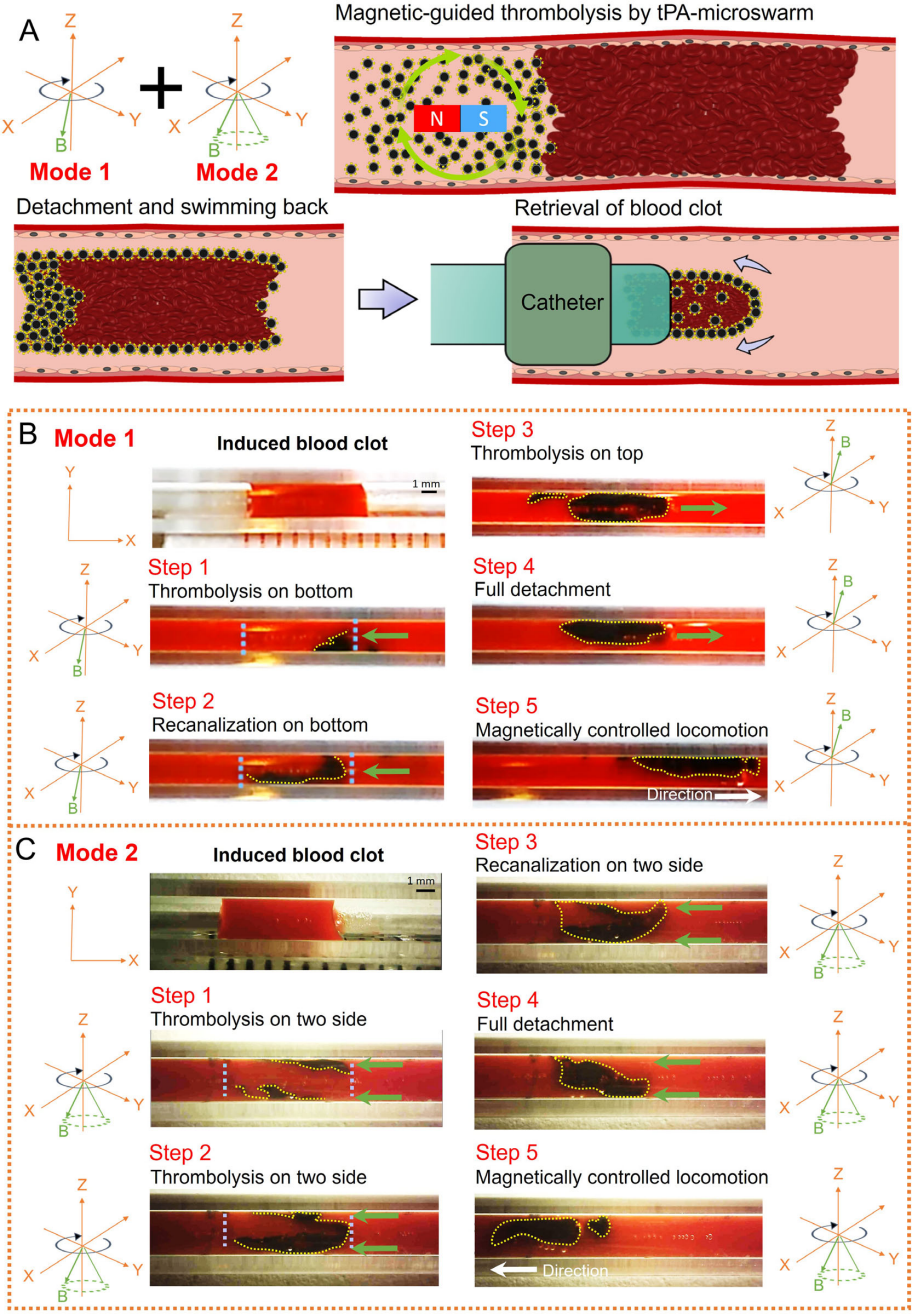
Fast detachment of the blood clot by two modes. A) Schematic of fast detachment and retrieval of the blood clot by applying two kinds of magnetic fields. B) Mode 1: Step 1, thrombolysis in the vessel bottom by applying the rotating magnetic field; Step 2, fast recanalization on the bottom; Step 3, the rotating magnetic field is adjusted to attract the tPA‐microswarm to the vessel top and degrade the blood clot; Step 4, The blood clot is fully detached from the vessel wall; Step 5, controllable locomotion of tPA‐microswarm together with magnetic debris for the further retrieval process. C) Mode 2: Step 1, conical rotating magnetic field enables simultaneous thrombolysis from two sides; Step 2, continuous thrombolysis from two sides; Step 3, fast recanalization from two sides; Step 4, The blood clot is fully detached from the vessel wall; Step 5, controllable locomotion of tPA‐microswarm together with magnetic debris for the further retrieval process. The green arrow refers to the thrombolysis direction.

Contrary to previous methods requiring complete clot removal and extended treatment time, here, we propose a novel strategy to restore the blood flow via the fast detachment of the blood clot using a tPA‐microswarm. **Figure** **3**A shows the schematic of the fast detachment and retrieval of the blood clot. tPA‐microswarm is directed to preferentially lyse the blood clot along the vessel wall by applying a designed magnetic field, aiding in the fast detachment of the blood clot. Here, two detachment modes are investigated and compared (see also Video S1, Supporting Information). For Mode 1 (Figure 3B), a rotating magnetic field with a downward gradient was applied, enabling tPA‐microswarm to lyse the blood clot along the bottom (Step 1). During the detachment process, tPA‐microswarm was able to penetrate and embed into the fibrin matrix, endowing the blood clot with magnetism. After recanalizing on the bottom, the magnetic field was adjusted to attract the tPA‐microswarm to the vessel top, which further degraded and detached the residual blood clot (Step 3). Once the blood clot is completely isolated from the vessel wall, tPA‐microswarm and blood clot debris can be navigated to the retrieval site controlled by the rotating magnetic field (Step 5). For Mode 1, recanalization was achieved in 10.5 min, suggesting this time‐efficient strategy could potentially enhance treatment outcomes for patients suffering from life‐threatening thrombotic disorders. The side view of the clot under Mode 1 treatment is also presented (Figure S5, Supporting Information), which clearly shows the clot transformation in the vessel throughout the thrombolysis and detachment process. Given varying application scenarios, providing a rotating magnetic field in the X‐Y plane (demonstrated in Mode 1) may be challenging due to spatial limitations. Hence, we also investigated using tPA‐microswarm for clot detachment from the side vessel wall with the rotating magnetic field along the X‐Z plane (Figure S6, Supporting Information). In Mode 1, the limited thickness of tPA‐microswarm restricts its function to essentially in‐plane thrombolysis, leading to the dissolution of the blood clot only along the vessel's bottom. By comparison, we verified that the clot lysis process could be achieved not only in one plane but also in the vertical direction by applying a conical rotating magnetic field as demonstrated in Mode 2. The tPA‐microswarm in Mode 2 transformed into a more dispersion state, and the thickness increased because the magnetic chains rotated obliquely upward instead of in‐plane rotation (Figure S7A (Supporting Information), Case 3). For Mode 1, the thickness of tPA‐microswarm was ≈0.54 mm (Figure S7A, (Supporting Information) Case 1); thus, the tPA‐microswarm mainly degraded the blood clot along the bottom due to the limited contact area. After applying a conical rotating magnetic field, both the thickness and contact area of the microswarm greatly improved. Compared to Mode 1, where the microswarm remains confined to the bottom surface of the vessel, Mode 2 enables interaction along both the bottom and vertical sides of the clot. In Figure S7 (Supporting Information), Case 3, a ≈1.8‐fold increase in the thickness of tPA‐microswarm (0.96 mm) is achieved compared with that of Mode 1, enabling the microswarm to lyse the blood clot vertically (along two sides of the vessel wall). After successfully separating the blood clot from the vessel wall, the tPA‐microswarm, together with the residual blood clot, was able to locomote to the targeted site for the retrieval process (Figure 3C, Step 5). Moreover, the simulation of the magnetic field generated by a 25 mm‐diameter permanent magnet was offered (Figure S8, Supporting Information). The microswarm is formed above the magnet tip, where the magnetic lines show a horizontal distribution. Moreover, as the increase of the distance between the microswarm and magnet tip, the magnetic field strength continuously decreases, which is highly consistent with our experimental results.

### 2.3 Recanalization Efficiency and NNPs Adhesion Analysis

**Figure 4:**
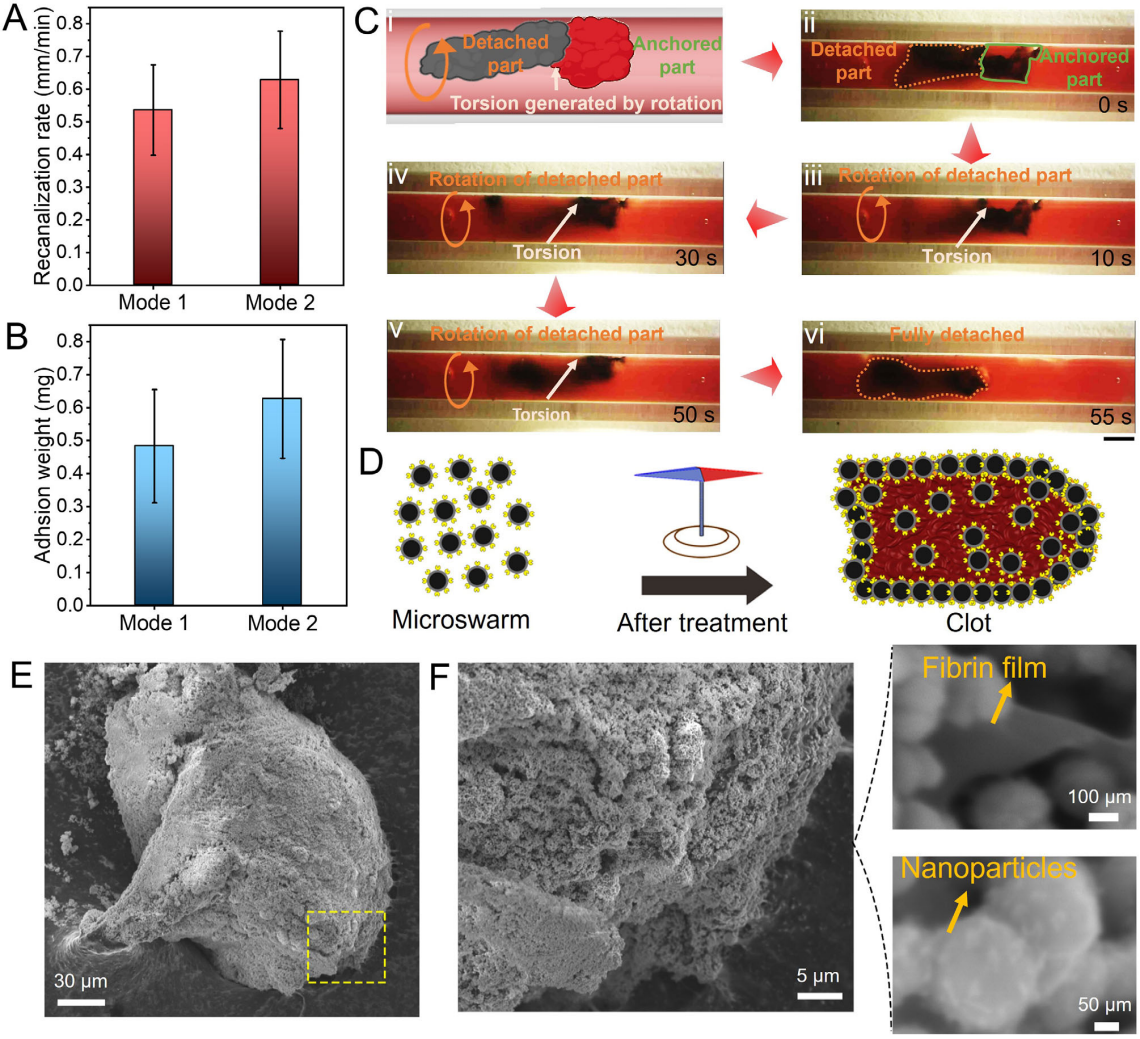
Recanalization efficiency and NNPs adhesion analysis. A) Quantitative analysis of recanalization efficiency for two detachment modes. B) The weight of NNPs adhesive to the blood clot after retrieval. C) Photo clips show the accelerated detachment process due to the torsion generated by the rotation of the detached blood clot (see also Video S3, Supplementary Video6). Scale bar, 1 mm. D) The schematic shows the magnetic debris formation process via the agglomeration of NNPs on the debris surface. (E‐F) Scanning electron microscope (SEM) images show the microstructure of the magnetic debris after treatment. The error bars donate the standard deviation (SD) from three experiments.

The quantitative result of the thrombolysis efficiency of the two modes was investigated (**Figure** **4**A). Both two modes show an advantage in fast recanalization, and the recanalization rate of Mode 1 is ≈0.54 mm min^−1^, while the recanalization rate of Mode 2 (0.63 mm min^−1^) is slightly higher than that of Mode 1. The modification of tPA on nanoparticles is critical for lysing and detaching the blood clot, and no obvious thrombolysis effect was observed using Fe_3_O_4_@SiO_2_ NNPs (Video S2, Supplementary Video2). Magnetizing the blood clot by tPA‐microswarm adhesion permits movability and further enables the retrieval process; thus, the state of adhesive tPA‐NNPs after treatment was analyzed. We re‐collected the detached blood clot and accessed the weight of tPA‐NNPs adhesive to the blood clot. Figure 4B shows that 0.48 mg of tPA‐NNPs adhered to the blood clot debris (Mode 1), which facilitates the fast detachment and retrieval process. By comparison, the weight of adhesive tPA‐NNPs increased to ≈0.63 mg in Mode 2 potentially due to the improvement of thickness and contact area of the microswarm under the conical rotating magnetic field. The results prove that the fast dissociation of the blood clot can be realized by the tPA‐microswarm via two modes, and effective adherence of magnetic particles to the blood clot allows controllable locomotion during the retrieval procedure.

Additionally, our investigation unveils that the separated segment of the blood clot is able to generate torsional force, thereby expediting the complete detachment of the blood clot from the vessel wall. During the degradation process, part of the blood clot can be first dislodged from the vessel wall with magnetic NNPs coated on its surface. Then the rotation of the detached part of the blood clot can induce the torsion force, accelerating the full detachment of the residual blood clot (Figure 4C(i)). A layer of NNPs coating on the detached blood clot can be observed with a black color (Figure 4C(ii)). The detached blood clot performed a rotation motion, which further generated the torsion force with the rotating magnetic field. The twisting force continuously tore the anchored part of the blood clot, accelerating the full detachment process of the blood clot. The blood clot was completely detached from the vessel wall within 1 min as shown in Figure 4C(vi) (see also Video S3, Supplementary Video3). As a result, the rotation motion of the separated blood clot could fasten the detachment process by the twisting force attributing to the effective adhesion of magnetic NNPs. After recanalization, the tPA‐microswarm together with the magnetic blood clot debris was collected to analyze the state of the detached blood clot. When applying a rotating magnetic field, the magnetic chains are formed by individual NNPs, and then those chains penetrate and embed inside the blood clot during the thrombolysis process. After treatment, the magnetic NNPs and detached blood clot debris form the agglomeration collective (Figure 4D). The SEM images in Figure 4E show the surface morphology of the blood clot debris consisting of substantial tPA‐NNPs. The microstructure of the blood clot debris after detachment is exhibited in Figure 4F, and tPA‐NNPs were distributed on the fibrin matrix of the blood clot debris. Those results indicate that magnetic tPA‐NNPs could effectively adhere to the blood clot matrix, which facilitates the detachment of the partly lysed blood clot and enables the retrieval process.

### 2.4 Controllable Thrombolysis with an Electromagnetic System

**Figure 5:**
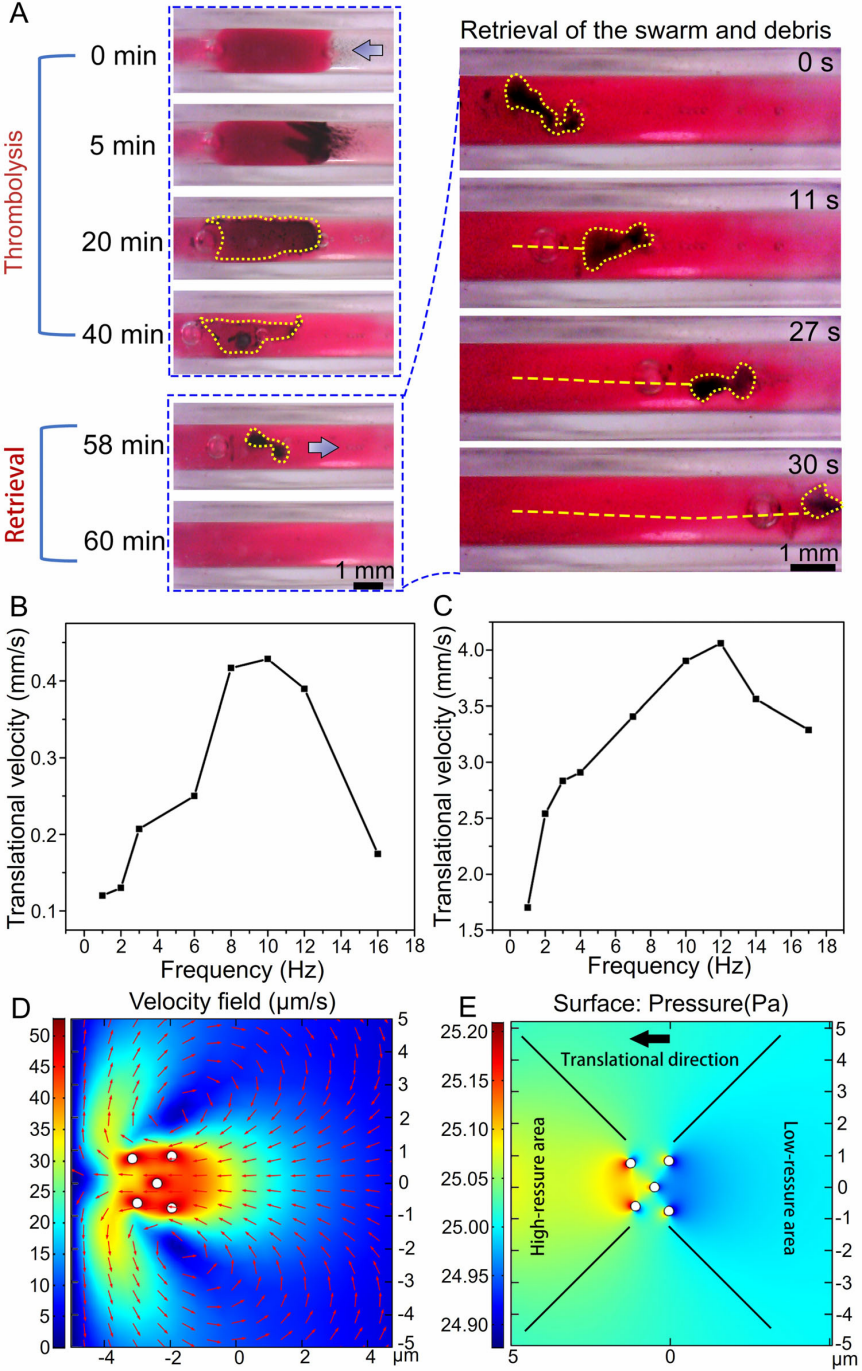
Controllable thrombolysis achieved by tPA‐microswarm with an electromagnetic system. A) Successive images show the uniform thrombolysis process. Magnetic field strength, 9 mT; frequency, 3 Hz; direction angle, 90 °C; (Video S4, Supplementary Video6). B) Relationship between the translational velocity of single nanocomposites and the input frequency. C) Relationship between the translational velocity of magnetic collectives and the input frequency. D) The simulation result shows the flow field of a cluster of NNPs rotating and translating toward a fixed boundary on the left side via the finite element method. E) The simulation result shows the pressure field of a cluster of NNPs rotating and translating toward a fixed boundary on the left side (the number in the color bar should plus 101300).

Compared with the permanent magnet‐based system, the electromagnetic system possesses advantages in flexible magnetic field design, fast on/off manipulability, simple current input strategy, etc., which offers opportunities to realize desirable actuation and motion control of magnetic objects in complex environments.^[^ ^13^ ^]^ Therefore, employing an electromagnetic system to achieve controllable thrombolysis and effective recanalization is also worth exploring. In order to better visualize the interaction between the tPA‐microswarm and the blood clot, the tube was filled with transparent PBS solution. tPA‐microswarm was able to homogeneously lyse the blood clot by applying a designed magnetic field (**Figure** **5**A). After 58 min, the majority of the blood clot was removed, and the residual blood clot coated with magnetic particles exhibited improved movability compared with individual tPA‐NNPs. The translational velocity of both the single NNPs and magnetic blood clot debris significantly increased with frequency reaching the plateau at 10 and 11 Hz, respectively (Figure 5B,C). By comparison, the maximal translational velocity of magnetic blood clot debris (4 mm s^−1^) is ≈9 times higher than single NNPs (0.45 mm s^−1^) due to its strong magnetism. The quantitative analysis of thrombolysis rates of pure tPA, Fe_3_O_4_ NNPs, and tPA‐microswarm was provided in Figure S9 (Supporting Information). The simulation of the thrombolysis process by the tumbling tPA‐microswarm was conducted by the finite element method. The movement of NNPs consists of self‐rotation and translation. Taking five particles as an illustration, self‐rotation results in the flow of local fluid near particles at the start moment (t = 0 s). Every particle moves with a rotational frequency of 3 Hz and a translational velocity of 50 µm s^−1^. When approaching the wall of the blood clot, fluid flows to the outer sides and scratches the wall (Figure 5D,E) effectively. In addition to uniform thrombolysis, one‐side thrombolysis can also be achieved and adopted in an emergency with its merits in faster running through the thrombus than uniform thrombolysis (Figure S10, Supporting Information). As a result, the uniform and effective separation of the blood clot was verified using an electromagnetic system with flexible and versatile magnetic field control, and the improved movability of detached magnetic debris contributed to the retrieval process.

### 2.5 Locomotion Ability and Micro‐CT Scanning of Detached Blood Clot Debris

**Figure 6:**
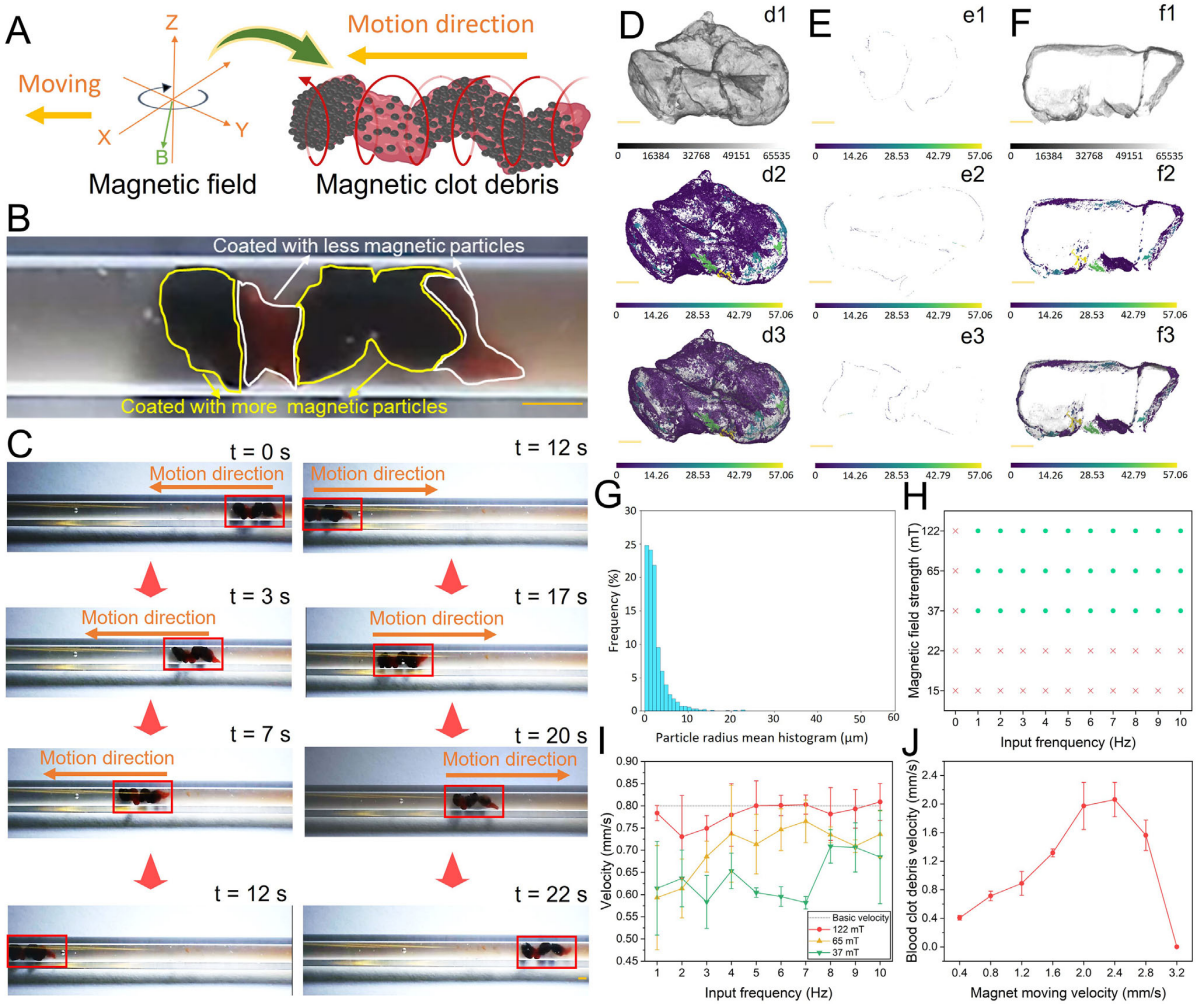
Locomotion ability and micro‐CT scanning of detached blood clot debris. A) Scheme of spiral locomotion of the blood clot debris with a rotating magnetic field. B) The distribution of the magnetic NNPs on the clot debris surface. C) Controllable locomotion of the blood clot debris in the tube. D) 3D scanning of the blood clot debris detached by Mode 1 (a1), 3D rendering of the blood clot debris after particle segmentation and extraction (a2), and overlap of 3D scanning and rendering of the blood clot debris (a3). E) Different orthogonal virtual slices of the blood clot debris along XY (b1), XZ (b2), and YZ (b3) direction. F) 3D internal structure of the blood clot debris after virtual cutting (c1) with 3D rendering (c2) and overlap (c3). G) The mean radius of magnetic particles on the blood clot debris detached by Mode 1. H) Phase diagram of the movability of the blood clot debris with various magnetic field strengths and frequencies, green dots indicate movability, and red crosses indicate non‐movability of the debris. I) The diagram shows the translational velocity of the clot debris with various magnetic field strengths and frequencies. J) The translational velocity of the clot debris with different moving speeds of the magnet. The scale bars in (B,C) are 1 mm, and the scale bars in (D–F) are 100 µm. The error bars donate the standard deviation (SD) from three experiments.

The movability of the blood clot debris after detachment was further investigated in the blood environment using a permanent magnet‐based system. As shown in **Figure** **6**A, the spiral motion of the magnetic blood clot can be realized by applying a rotating magnetic field. Figure 6B shows optical imaging of the detached blood clot coated with magnetic particles, and the area circled by yellow lines was coated with more NNPs compared with the area circled by white lines. With the rotating magnetic field, the blood clot debris performed a spiral motion gait, and controllable locomotion was achieved by continuously moving the sphere magnet that generated a magnetic gradient along the moving direction. As shown in Figure 6C, forward and backward locomotion were realized by adjusting the moving direction of the magnet. The locomotion ability of detached blood clots with random shapes was further confirmed (Video S6, Supplementary Video6), and all 8 samples were able to perform controllable locomotion, suggesting our reliable and effective control strategy. To determine the 3D structure of the detached blood clot debris and magnetic NNPs distribution, micro‐CT scanning was performed, as shown in Video S7 (Supplementary Video7). As shown in Figure 6D(d1), the detached blood clot showed a cylinder‐like shape with magnetic NNPs coating. Then, the 3D rendering of the particle distribution was offered after the segmentation and extraction process, revealing that the majority area of the detached blood clot debris was coated by magnetic particles. Moreover, the overlap of 3D scanning and rendering of the blood clot debris suggested that the particle distribution on the clot surface was uneven. Different orthogonal virtual slices of the blood clot debris along XY, XZ, and YZ directions were provided (Figure 6E(e1‐e3)), which indicated the thickness of the coated particles ranging from 0 to 28.3 µm. The internal structure of the blood clot after virtual cutting was presented in Figure 6F, demonstrating that magnetic particles were not only adhesive to the blood clot surface but also penetrated inside of clot debris. As depicted in Figure 6G, the mean radius was measured to determine the aggregation state of the magnetic NNPs. The result verified that the majority of magnetic particles possessed a mean radius of less than 10 µm, indicating that those particles maintained a relatively dispersive state instead of forming larger clusters. We obtained 8 samples of detached blood clots, and the 3D structures and particle distributions can be found in Figures S11 and S12 (Supporting Information). Those samples exhibited random shapes with sufficient magnetic particle coating.

After confirming the magnetic particle distribution, the quantitative analysis of the locomotion ability of the blood clot debris was investigated. A phase diagram of the movability of the blood clot debris with various magnetic field strengths and frequencies was depicted in Figure 6H. To enable the locomotion of the blood clot, the input frequencies of the rotating magnetic field should be higher than 1 Hz, while the lowest magnetic field strength is 37 mT. The locomotion velocity of the blood clot was further analyzed with various experimental parameters (Figure 6I). Here, the basic velocity indicated the moving velocity of the sphere magnet (0.8 mm s^−1^). With the magnetic field strength of 122 mT, the average translational velocity of the blood clot fluctuated from 0.70 to 0.8 mm s^−1^, and the velocity maintained ≈0.8 mm s^−1^ when input frequencies were larger than 5 Hz. For magnetic field strength decreased to 65 mT, the average translational velocity gradually increased from 0.59 to 0.74 mm s^−1^ with input frequency improving from 1 to 5 Hz. Once the input frequency exceeded 7 Hz, the average translational velocity started to decrease due to the step‐out frequency effect. As the magnetic field strength decreased to 37 mT, a sharp drop in the velocity can be observed. We further investigated the influence of the moving velocity of the magnet on the translational velocity of the detached blood clot. As shown in Figure 6J, the average translational velocity of the blood clot increased when the moving velocity of the magnet was smaller than 2.4 mm s^−1^, then dramatically decreased to 0 mm s^−1^ at the moving velocity of 3.2 mm s^−1^. Therefore, to maintain effective locomotion of the blood clot, the maximal moving velocity of the magnet was 2.8 mm s^−1^. Our results proved that controllable locomotion of the detached blood clot debris can be achieved in a wide parameter range of the rotating magnetic field.

Moreover, we also explored the relationship between clot mass, magnetic field strength, and the movability of clot debris, which can benefit the design and optimization of magnetic‐based interventions for the treatment of blood clots, where the ability to manipulate and remove clot debris is crucial (Figure S13, Supporting Information). When the magnetic clot is weighted smaller than 21.10 mg, controllable locomotion can be achieved even with the magnetic field strength of 8 mT. Moreover, as the clot mass increases, stronger magnetic field strengths are required to guarantee the movability of the clot debris. As the mass of the clot was located at the range of 42.20 to 63.30 mg, the clot debris maintained the movability with the magnetic field strength larger than 22 mT, suggesting that effective locomotion of the clot debris could be realized via adjusting the input magnetic field strengths.

Furthermore, the content of NNPs is another important factor determining the movability of the clot debris, and a comprehensive evaluation was offered (Figure S14, Supporting Information). When the content of NNPs was lower than 0.02 mg, the clot debris showed non‐movability. As the content of NNPs increased, the movability of the clot gradually enhanced, and the required magnetic field strengths that ensured the effective locomotion decreased correspondingly. The threshold value of the magnetic field strength that allowed motion control of the clot was 8 mT. At a typical range of 0.20 to 0.8 mg, the minimized magnetic field strength enabling locomotion of clot was 37 mT. Such exploration provided necessary references to ensure optimal operation conditions for the clot debris with various magnetic field strengths and NNPs contents.

### 2.6 Fast Recanalization and Remote Retrieval Process in the Phantom

**Figure 7:**
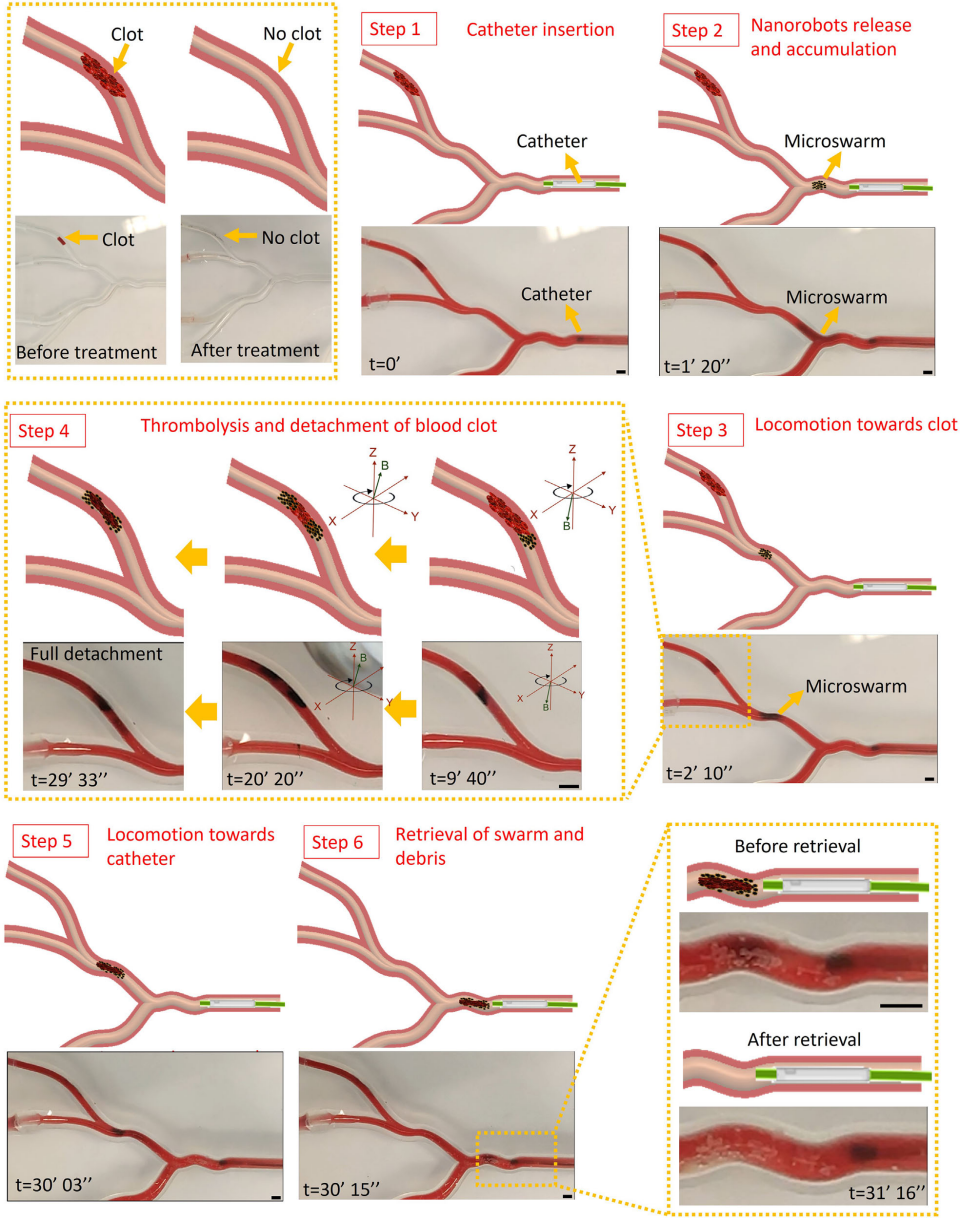
Fast recanalization and remoted retrieval process in the phantom with multiple narrow branches. Step 1, catheter insertion process; Step 2, nanorobots release and accumulation process; Step 3, controllable locomotion of tPA‐microswarm toward the blood clot; Step 4, thrombolysis and detachment of the blood clot; Step 5, controllable locomotion of tPA‐microswarm toward the catheter; Step 6, retrieval of tPA‐microswarm and magnetic debris (Video S8, Supplementary Video8). All scale bars, 3 mm.

The demonstration in the phantom with multiple vessel branches and dynamic flow was conducted to evaluate the validity of our fast recanalization strategy in a complex environment. A blood clot with a length of 5 mm was generated in one branch of the phantom, where the branch was too narrow for the catheter to reach. The whole phantom was immersed in a tank filled with water at 37 °C to mimic human body temperature. The catheter insertion process is shown in **Figure** **7**, Step 1, and the tPA‐NNPs were delivered to the region close to the branch blocked by the blood clot. An advantage of the catheter‐based delivery strategy is that it can minimize the potential loss of those therapeutic agents during the delivery process in the vessel even with high blood flow. After arriving at the targeted site, 1 mg of tPA‐NNPs were released from the catheter and were trapped by the rotating magnetic field to form the microswarm (Step 2). Then, tPA‐microswarm was navigated to the branch with the blood clot for the targeted thrombolysis and detachment process (Step 3). tPA‐microswarm first degraded the blood clot from the bottom, and the distribution of tPA‐microswarm around two sides of the blood clot indicated that recanalization was realized on the bottom (Step 4, middle photograph). Then the rotating magnetic field was adjusted to enable tPA‐microswarm to lyse the blood clot on the vessel top, achieving full detachment of the residual blood clot after ≈20 min thrombolysis. Thanks to its excellent magnetism, the detached blood clot debris was able to locomote toward the catheter tip guided by the rotating magnetic field (Step 5). After arriving at the retrieval site, the tPA‐microswarm and magnetic debris were retrieved by catheter suction, and no residuals were observed after the retrieval procedure (Step 6). Our results confirmed the feasibility of using a catheter to accomplish the retrieval of therapeutic agents and blood clot fragments after rapid detachment. This method could reduce possible side effects and prevent secondary blockages in narrow distal blood vessels, showing great potential for the treatment of time‐dependent thrombolytic disorders. Moreover, the system retrieval efficiency was quantitively investigated in the phantom, and the result showed that 90.67% ± 4.73% of magnetic particles can be retrieved from the vessel, suggesting our highly effective recovery strategy for magnetic particles.

### 2.7 Reducing the Blood Flow in an Ex Vivo Model

It is a great challenge that the strong drag force induced by high blood flow may disturb the stability and locomotion ability of the tPA‐microswarm, especially in the cerebral vascular where the blood flow can be as high as 20 cm ^−1^s, leading to treatment failure. To overcome such an issue, we further adapted the catheter balloon to decrease the blood flow, which ensures the movability and retrieval of the tPA‐microswarm and clot debris, using the human placenta as the model. Figure S15A (Supporting Information) shows the placenta with selected blood vessels used in our experiment, while the balloon catheter intervention can be performed as shown in the last picture. To verify the feasibility of the catheter balloon in reducing blood flow, laser speckle contrast imaging (LSCI) was employed to measure the blood flow change with or without the catheter balloon expansion. Six typical regions were used as the indicators to evaluate the perfusion unit (PU) variation as shown in Figure S15B (Supporting Information). Significant imaging signal reduction can be observed after balloon catheter intervention both in the pseudo color patterns and gray patterns. The quantitative result of the PU change showed that ≈85.96%, 80.104%, 90.46, 67.29%, 85.51%, and 91.05% reductions in the PU value were achieved for various regions. The diameter of the blood vessels at different positions was measured (Figure S15E, Supporting Information), and the flow velocity in position 3 decreased from 18.20 to only 0.56 cm ^−1^s after catheter balloon expansion. Those results firmly verified that balloon catheter intervention was an effective approach to reduce the high blood flow, benefitting the retrieval process. Furthermore, the demonstration of retrieval of the tPA‐microswarm and clot debris was performed in the placenta with catheter balloon intervention. Figure S15G (Supporting Information) showed the whole procedure, including catheter balloon intervention, controllable locomotion of tPA‐microswarm, clot delamination, controllable locomotion of tPA‐microswarm and clot debris, as well as retrieval process, proving the feasibility of the proposed strategy to decrease the blood flow and guarantee the retrieval procedure via effective and viable balloon expansion.

### 2.8 Fast Recanalization and Remote Retrieval Process in the Pig Model

**Figure 8:**
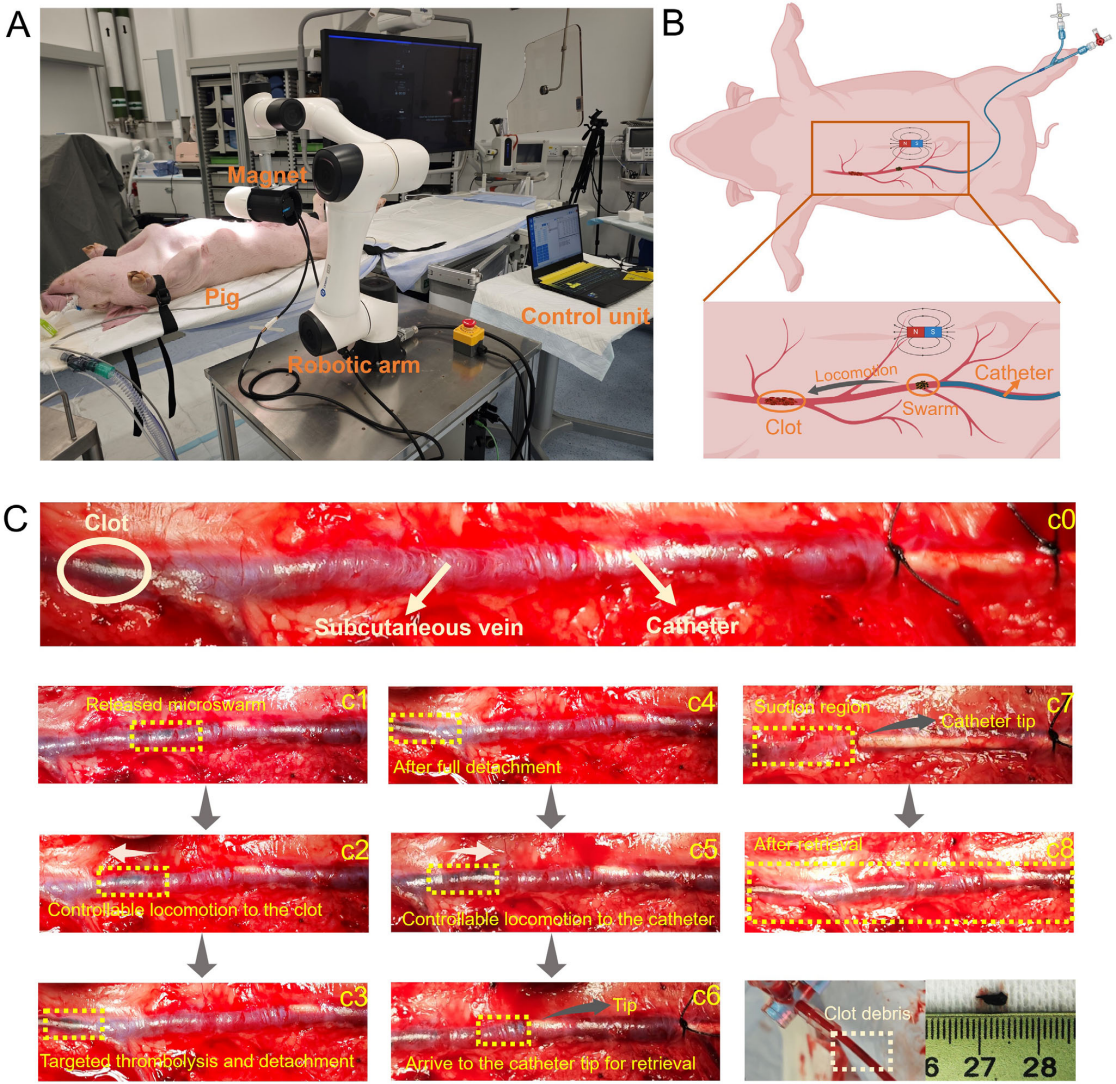
In vivo thrombolysis and debris retrieval using tPA‐functionalized magnetic microswarm in a live pig model. A) Experimental setup showcasing the robotic arm with a magnet, control unit, and catheter system for guiding the microswarm in the subcutaneous vein of the pig's abdominal region. B) Schematic representation of the experimental workflow, including clot localization, microswarm locomotion, thrombolysis, and debris retrieval. Schematic was created using BioRender.com. C) (c0–c8) Real‐time images of the thrombolysis process: (c0) Initial clot observed in the subcutaneous vein. (c1) Release of the microswarm from the catheter. (c2) Controlled locomotion of the microswarm to the clot site. (c3) Targeted thrombolysis and clot detachment. (c4) Complete clot detachment. (c5) Controlled locomotion of the microswarm to the catheter tip. (c6) Microswarm arriving at the catheter tip for retrieval. (c7) Clot debris suctioned into the catheter. (c8) Retrieved clot debris.

The feasibility of the proposed strategy that achieves rapid blood clot removal via remote delamination and magnetization of clot debris was further verified in vivo. An adult pig was employed as the living animal model as illustrated in **Figure** **8**A, and the microswarm successfully demonstrated precise navigation to the clot site within the subcutaneous vein, efficient thrombolysis, and subsequent retrieval of clot debris. The actuation system was assembled on the robotic arm for feasible manipulation by the control unit. The subcutaneous abdominal vein of the pig was selected as the vascular model (Figure 8B), and the clot was positioned away from the catheter tip. A microcatheter with an inner diameter of 2.5 mm was used for tPA‐microswarm delivery and retrieval of the clot debris, and the locomotion of the microswarm was controlled by the magnetic actuation system. The process, guided by an external magnetic field, included targeted clot detachment, controlled microswarm locomotion back to the catheter tip, and debris suction into the catheter. Figure 8C(c0) shows the real photo of the subcutaneous abdominal vein of the pig after surgical exposure, and the blood clot model was constructed inside the blood vessel. The microcatheter was inserted into the blood vessel and temporarily fixed with surgical sutures. The tPA‐NNPs were delivered and released from the catheter (Figure 8C(c1)), and then tPA‐NNPs were trapped by the magnetic field and formed the microswarm as circled by the yellow rectangle. Subsequently, controllable locomotion of the tPA‐microswarm was performed with the manipulation of the magnetic actuation system, while the microswarm maintained a stable and collective state during the moving procedure (Figure 8C(c2)). Once the tPA‐microswarm arrived at the clotting region, effective thrombolysis was conducted to enable fast detachment and magnetization of clot debris (Figure 8C(c3)). After full delamination, the microswarm together with the clot debris was magnetically controlled to move to the catheter tip for the retrieval procedure (Figure 8C(c5,c6)). The blood vessel became flattened with the catheter suction process, and both the microswarm and clot debris were retrieved from the blood vessel, with no obvious residuals observed after the retrieval procedure (Figure 8C(c8)). The retrieved clot debris was further re‐collected as shown in the last photo of Figure 8C, showing a diameter of ≈2 mm and a length of ≈5 mm. The successful demonstration verified the feasibility of the proposed strategy, paving the way for future pre‐clinical and/or clinical translation of functional microswarms tailored for thrombus treatment, benefiting the patients.

### 2.9 In Vivo Safety Evaluation

To assess the in vivo safety of tPA‐labeled microswarms following intravenous injection, we conducted a comprehensive blood analysis. Rats were divided into three groups: a control group receiving 1 mL of PBS, and two experimental groups receiving 0.1 and 0.5 mg of tPA‐labeled NNPs, respectively. Blood samples were collected on day 7 for analysis. As illustrated in Figure S16 (Supporting Information), the levels of blood cells (including red blood cells, white blood cells, and platelets) and biochemical markers in the experimental groups remained within normal ranges, showing only minor fluctuations compared to the control group. These findings demonstrate that tPA‐labeled microswarms exhibit a favorable in vivo safety profile across different concentrations, with no significant adverse effects observed even over an extended period. This evaluation confirms the therapeutic potential of tPA‐labeled microswarms and highlights their minimal side effects on biological systems.

## 3 Conclusion

In conclusion, our work presents a novel strategy to remove the blood clot for time‐saving vascular recanalization using a remote retrieval method. The blood clot was partly degraded by tPA‐microswarm, and rapid detachment of blood clot debris from the vessel wall was achieved by applying the designed magnetic field. Two detachment modes were explored for fast recanalization with detachment efficiency of 0.52 and 0.61 mm min^−1^ for Mode 1 and Mode 2, respectively. The effective degradation and detachment process was further verified by an electromagnetic system featuring with programmable design of magnetic field and flexible motion control. The detached blood clots with random shapes and uneven particle distribution possess excellent locomotion ability with the rotating magnetic field. Moreover, such a strategy has been successfully validated in a phantom model with narrow and tortuous branches, and the whole treatment procedure can be completed within 32 min, involving catheter delivery, controllable locomotion, fast detachment, and retrieval process. Moreover, the demonstration was successfully conducted in vivo using a living pig model. Taken together, the strategy we proposed that can shorten the recanalization time by the fast detachment of the blood clot and minimize the side effects endowed by the catheter retrieval procedure, is promising for strengthening treatment outcomes of thrombolytic disorders, such as ischemic stroke.

By comparison, previous micro/nanorobots were commonly used as drug carriers enabling localized therapy for removing blood clots.^[^ ^4^, ^14^ ^]^ On one hand, to fully degrade the blood clots, a long treatment duration is needed, which may attenuate the therapeutic outcome for patients, considering the nerve tissue necrosis caused by prolonged hypoxia; On the other hand, regeneration of blood clot may occur if the residual blood clot still adheres to the blood vessel wall, and potentially formed blood clot debris during the treatment may induce secondary occlusion with bloodstream. To tackle those issues, a microswarm‐catheter hybrid system was developed to achieve fast detachment and retrieval of the blood clot. By applying a designed magnetic field, tPA‐microswarm was able to degrade the blood clot on‐demand, which could detach the blood clot from the vessel wall effectively and accelerate recanalization. During the thrombolysis, substantial magnetic nanoparticles were coated on the blood clot; thus, the detached blood clot can be magnetically navigated to the catheter for further retrieval process, avoiding secondary occlusion in narrow distal blood vessels.

Although the validity of the controlled blood clot retrieval strategy has been confirmed in an artificial blood vessel model and the subcutaneous vein of the pig, some challenges should be overcome in our future work. 1) To realize the practical and clinical application of the magnetic microswarm‐catheter system, a combination of imaging modalities is necessary. Visualizing the microswarm can not only strengthen the delivery efficiency but also ensure the biosafety of those administrated therapeutic agents in the human body. Therefore, employing proper imaging tools, such as fluoroscopy, magnetic resonance imaging (MRI),^[^ ^15^ ^]^ ultrasound (US) imaging,^[^ ^6^, ^14^ ^]^ and photoacoustic imaging (PAI),^[^ ^16^ ^]^ to integrate with our proposed microswarm‐catheter system should be considered in our future work. 2) A real in vivo environment is dynamically changing, while uncontrollable factors are much more difficult to predict and handle in such a complex environment. Therefore, the evaluation of our fast detachment and retrieval strategy needs to be further conducted in vivo with artery models (e.g., the cerebral model) featuring high blood flow and narrow vascular distribution, which could prompt the development of micro/nanorobotic platforms for practical stroke treatment.

## 4 Experimental Section

### Materials

Bis‐NHS‐PEG6, sodium citrate, Tetraethyl orthosilicate (TEOS), FeCl_3_·6H_2_O (iron (III) chloride hexahydrate), CaCl_2_ (calcium chloride), dimethyl sulfoxide (DMSO), 1‐octadecene, hexadecyltrimethylammonium chloride (CTAC), and ethylene glycol (EG) were purchased from Aladdin Chemical Co., Ltd. Polyethylene glycol (PEG), C_2_H_3_NaO_2_·3H_2_O (sodium acetate trihydrate), (3‐Aminopropyl) triethoxysilane (APTES) were purchased from Sigma‐Aldrich. All of the chemicals were used without further purification.

### Synthesis of tPA‐nanorobots

Spherical Fe_3_O_4_ NNPs with a diameter of 300–400 nm were prepared by a hydrothermal method. Briefly, 1 g of FeCl_3_·6H_2_O was added into the beaker with 40 mL of EG followed by mechanical stirring to fully dissolve the FeCl_3_·6H_2_O. Next, 2.7 g of C_2_H_3_NaO_2_·3H_2_O was dissolved in EG with continuous stirring for 10 min. Then, 0.75 g of PEG was mixed with EG solution and stirred overnight for complete dissolving. Subsequently, the mixture was transferred into the Teflon‐autoclave and heated for 10 h at 200 °C. After cooling to room temperature, the magnetic black particles were collected by a magnet and washed with ethanol and DI water 3 times. The product was stored with a concentration of 10 mg mL^−1^ for further use.

A thin layer of mesoporous SiO_2_ was coated on the surface of the Fe_3_O_4_ NNPs via a biphasic stratification reaction. 3 g of CTAC and 40 mg of Fe_3_O_4_ NNPs were dissolved in a round‐bottomed flask with 60 mL of water and then stirred in a water bath with sonication at 60 °C to fully disperse the Fe_3_O_4_ NNPs. Then, 160 µL of TEA was added. Next, 20 mL of 1‐octadecene with 10% v/v% TEOS solution was added into the flask 3 times with a time interval of 10 min. The reaction was continued for 15 h, and the final production (Fe_3_O_4_@SiO_2_) was collected by a magnet and washed with ethanol and DI water 3 times.

Fe_3_O_4_@SiO_2_ NNPs were then mixed with 1 mL of APTES in 50 mL of ethanol for surface grafting. After 12 h, APTES grafted Fe_3_O_4_@SiO_2_ NNPs were harvested and washed with DMSO 3 times. Then 20 mg of Fe_3_O_4_@SiO_2_ NNPs were added into the DMSO (10 mL) solution with 1 mg mL^−1^ of Bis‐NHS‐PEG_6_ and 1.7 mM of triethylamine, aging for 2 h at room temperature. After aging, Fe_3_O_4_@SiO_2_ NNPs were washed with DMSO and PBS (pH 7.2) 3 times. Afterward, Fe_3_O_4_@SiO_2_ NNPs were mixed with tPA (2.5 mg mL^−1^) in 20 mL of PBS solution, reacting for 2 h with stirring. Finally, tPA‐nanorobots were successfully prepared and stored in PBS solution with a concentration of 1 mg mL^−1^ at 4 °C.

### Enzyme Activity Test

The enzyme activity of prepared tPA‐NNPs was assessed via the substrate chromogenic method. The tPA Human Chromogenic Activity Assay Kit was used to determine the tPA activity in cell culture supernatants, which contained the plasminogen, plasmin substrate, and tPA, respectively. To evaluate the enzyme activity, the microplate reader was used to measure the absorbance of various samples at 405 nm with an incubation temperature of 37 °C. 50 µL of plasminogen solution was mixed with equal volume of plasmin substrate in the 96‐well plate, then 0.1 mg of prepared tPA‐NNPs and 0.001 mg of pure tPA were added into separated wells with gentle agitation. Then the wells were sealed with pressure‐sensitive sealing tapes and placed in the microplate reader, the incubation temperature was 37 °C. The OD values at 405 nm were recorded at various time intervals.

### Blood Clot Inducing in Tube and Phantom

The blood clot was induced by using CaCl_2_ solution as the triggering agent. 1 mL of fresh pig blood was first mixed with 20 µL of CaCl_2_ (0.5 m), then the pig blood was injected into the tube or the phantom branch with a desired length (typically 5–7 mm) followed by incubation for 20 min at 37 °C. After successfully forming the blood clot, pure pig blood was filled into the tube and phantom.

### Blood Clot Detachment in the Tube

After inducing the blood clot in the tube, 1 mg of tPA‐NNPs was injected into the tube, and a rotating magnetic field with a frequency of 3 Hz and magnetic field strength of 64.9 mT was applied, where the N‐S poles of the magnet (diameter, 25 mm) were on X‐Y plane. Then, the tPA‐microswarm was formed and navigated to the blood clot for thrombolysis. For Mode 1, the rotating sphere magnet was applied from the vessel bottom, then adjusted above the vessel top to realize full detachment of the blood clot. For Mode 2, the conical rotating magnetic field was generated to achieve simultaneous thrombolysis from two sides, where the N‐S poles of the magnet show a pitch angle of 45°. For the electromagnetic system, the magnetic field strength was 9 mT, the frequency was 3 Hz, and the direction angle was 90°.

### Demonstration in the Phantom

The phantom with multiple branches was immersed in the tank filled with circulating hot water to maintain a constant temperature of 37 °C. The diameter of the phantom branch with the induced blood clot was 1.5 mm. A catheter (Advance 35LP) with PTA balloon dilatation was used in the experiment. The blood (4X diluted) was circled by using a peristaltic pump. The catheter was deployed into the phantom at a distance of ≈45 mm from the blood clot. Then the tPA‐NNPs (2 mg mL^−1^) were released from the catheter with a total dose of 1 mg; meanwhile, the blood flow was temporarily blocked by the catheter balloon. Then those accumulated NNPs formed the tPA‐microswarm with the rotating magnetic field (frequency, 3 Hz; magnetic field strength, 37.2 mT). The tPA‐microswarm was steered and navigated to the blood clot site (distance of 5.5 cm) for the thrombolysis and detachment process. The tPA‐microswarm first degraded the blood clot on the bottom, and then the rotating magnetic field was adjusted to enable tPA‐microswarm to lyse the blood clot on the vessel top. After full detachment, the detached blood clot debris and the residual tPA‐microswarm were navigated toward the catheter tip. After arriving at the catheter site, the nanocomposite collectives and magnetic debris were retrieved by catheter suction.

### Ex Vivo Experiment

The human placenta sourced from the Prince of Wales Hospital was utilized for ex vivo studies. The collection process was sanctioned and monitored by The Joint Chinese University of Hong Kong–New Territories East Cluster Clinical Research Ethics Committee (reference number 2020.384). All participating patients gave their written informed consent. After collection, the human placenta was washed with fresh saline with a concentration of 0.9 wt.%, and the placental membrane was carefully removed to avoid damaging the blood vessels. Then, the saline was injected into the blood vessel to clear the residual blood. After that, the blood vessels were connected with silicone tubes to enable free flow, and the flow velocity was controlled by the peristaltic pump. First, the blood clot was induced in one branch of the blood vessel. 20 µL of 0.5 m CaCl_2_ solution was added to 1 ml of fresh pig blood, and, then, the blood was injected into the tube and placed in the oven for incubation at a temperature of 37 °C. After 20 min, the generated blood clot was injected into the placenta, and the placenta was observed by LSCI. The dosage of the tPA‐microswarm used in the experiment was 1 mg, and input frequency and magnetic field strength were 4 Hz and 60 mT, respectively.

### In Vivo Experiment

The living animal experiment was carried out in accordance with the guidelines of the Animals Ordinance (Chapter 340), and approved by the Animal Experimentation Ethics Committee (no. 22–1036) in the Chinese University of Hong Kong. Before the experiment, the anesthesia was performed by injecting a mixture of atropine, xylazine, and ketamine into the pig. Then, anesthesia was continued by using 4% pentobarbitone, while a 1:1 mixture of 2% isoflurane to oxygen and nitrous oxide was supplied at a rate of 5 L min^−1^. Then, the subcutaneous abdominal vein of the pig was surgically exposed, followed by microcatheter insertion with an inner diameter of 2.5 mm. To construct the blood clot model, the thrombus with an initial diameter of 2 mm and a length of 6 mm, was first generated in the incubator at a temperature of 37 °C, and then was injected into the desired position inside of the pig blood vessel away from the catheter. 1 mg of tPA‐NNPs were delivered and released via catheterization, with continuous magnetic actuation. The parameter of the magnetic field was determined with the input frequency of 4 Hz and a magnetic field strength of ≈60 mT to ensure robust and effective actuation and manipulation.

### Characterization and Measurements

SEM images were captured by using a JEOL JSM‐7800F scanning electron microscope. TEM images were obtained using the JEOL Model JEM‐2011 System. Zeta potential and DLS tests were performed by particle analyzer (ZS90, LC10‐HS). XRD spectrum was measured by an X‐ray diffractometer (Bruker D8 Advance). The enzyme activity test was conducted by a microplate reader (Infinite M PLEX, TECAN).

## Conflict of Interest

The authors declare no conflict of interest.

## Author Contributions

Q.L.W., B.W., and L.Z. performed conceptualization. Q.L.W., B.W. performed methodology. Q.L.W., B.W., K.F.C., X.S., Q.Q.W, F.T.J., L.S., B.Y.M.I., H.K., P.W.Y.C., T.W.H.L., and L.Z. performed investigation. Q.L.W. and B.W. performed visualization. B.W., L.Z. performed supervision. Q.L.W. wrote the original draft. Q.L.W., B.W., and L.Z. wrote, reviewed, and edited the original draft.

## Supporting information



Supporting Information

Supplementary Video1

Supplementary Video2

Supplementary Video3

Supplementary Video4

Supplementary Video5

Supplementary Video6

Supplementary Video7

Supplementary Video8

## Data Availability

The data that support the findings of this study are available in the supplementary material of this article.

## References

[1] a) A. Zenych , L. Fournier , C. Chauvierre , Biomaterials 2020, 258, 120297; 32818824 10.1016/j.biomaterials.2020.120297

[2] a) A. V. Alexandrov , A. M. Demchuk , W. S. Burgin , D. J. Robinson , J. C. Grotta , J. Neuroimag. 2004, 14, 113; 15095555

[3] S. Hassanpour , H.‐J. Kim , A. Saadati , P. Tebon , C. Xue , F. W. van den Dolder , J. Thakor , B. Baradaran , J. Mosafer , A. Baghbanzadeh , N. R. de Barros , M. Hashemzaei , K. J. Lee , J. Lee , S. Zhang , W. Sun , H.‐J. Cho , S. Ahadian , N. Ashammakhi , M. R. Dokmeci , A. Mokhtarzadeh , A. Khademhosseini , Small 2020, 16, 2001647. 10.1002/smll.202001647 PMC7702193 32790000

[4] a) J. Hu , S. Huang , L. Zhu , W. Huang , Y. Zhao , K. Jin , Q. ZhuGe , ACS Appl. Mater. Interfaces 2018, 10, 32988; 30192506 10.1021/acsami.8b09423

[5] a) H. Ceylan , I. C. Yasa , U. Kilic , W. Hu , M. Sitti , Prog. Biomed. Eng. 2019, 1, 012002;

[6] a) B. Wang , K. F. Chan , K. Yuan , Q. Wang , X. Xia , L. Yang , H. Ko , Y.‐X. J. Wang , J. J. Y. Sung , P. W. Y. Chiu , L. Zhang , Sci. Robot. 2021, 6, eabd2813; 34043547 10.1126/scirobotics.abd2813

[7] a) J. R. Baylis , J. H. Yeon , M. H. Thomson , A. Kazerooni , X. Wang , A. E. St. John , E. B. Lim , D. Chien , A. Lee , J. Q. Zhang , J. M. Piret , L. S. Machan , T. F. Burke , N. J. White , C. J. Kastrup , Sci. Adv. 2015, 1, e1500379; 26601282 10.1126/sciadv.1500379 PMC4646796

[8] a) J. Wang , R. Dong , Q. Yang , H. Wu , Z. Bi , Q. Liang , Q. Wang , C. Wang , Y. Mei , Y. Cai , Nanoscale 2019, 11, 16592; 31460538 10.1039/c9nr04295d

[9] a) B. E.‐F. de Ávila , P. Angsantikul , J. Li , M. A. Lopez‐Ramirez , D. E. Ramírez‐Herrera , S. Thamphiwatana , C. Chen , J. Delezuk , R. Samakapiruk , V. Ramez , M. Obonyo , L. Zhang , J. Wang , Nat. Commun. 2017, 8, 272; 28814725 10.1038/s41467-017-00309-w PMC5559609

[10] a) Y. Wang , Y. Liu , Y. Li , D. Xu , X. Pan , Y. Chen , D. Zhou , B. Wang , H. Feng , X. Ma , Research 2020, 2020, 7962024; 32566931 10.34133/2020/7962024 PMC7293755

[11] a) A. C. Hortelão , T. Patiño , A. Perez‐Jiménez , À. Blanco , S. Sánchez , Adv. Funct. Mater. 2018, 28, 1705086;

[12] Q. Wang , B. Wang , J. Yu , K. Schweizer , B. J. Nelson , L. Zhang , in 2020 IEEE International Conference on Robotics and Automation (ICRA) 2020, pp. 10285–10291.

[13] a) I. C. Yasa , H. Ceylan , U. Bozuyuk , A.‐M. Wild , M. Sitti , Sci. Robot. 2020, 5, eaaz3867; 33022620 10.1126/scirobotics.aaz3867

[14] a) L. Wang , J. Wang , J. Hao , Z. Dong , J. Wu , G. Shen , T. Ying , L. Feng , X. Cai , Z. Liu , Y. Zheng , Adv. Mater. 2021, 33, 2105351; 10.1002/adma.202105351 34647345

[15] a) G. Go , A. Yoo , K. T. Nguyen , M. Nan , B. A. Darmawan , S. Zheng , B. Kang , C.‐S. Kim , D. Bang , S. Lee , K.‐P. Kim , S. S. Kang , K. M. Shim , S. E. Kim , S. Bang , D.‐H. Kim , J.‐O. Park , E. Choi , Sci. Adv. 2022, 8, eabq8545; 36399561 10.1126/sciadv.abq8545 PMC9674283

[16] Z. Wu , L. Li , Y. Yang , P. Hu , Y. Li , S.‐Y. Yang , L. V. Wang , W. Gao , Sci. Robot. 2019, 4, eaax0613. 32632399 10.1126/scirobotics.aax0613 PMC7337196

