# Supplementary material for: Rapid Blood Clot Removal via Remote Delamination and Magnetization of Clot Debris
Source: Adv Sci (Weinh). 2025 Mar 8;12(17):2415305. doi: 10.1002/advs.202415305 (PMC12061269; doi:10.1002/advs.202415305)
Supplement: Supplementary file 1 — Supporting Information [file ADVS-12-2415305-s007.docx]

Supporting Information

**Rapid Blood Clot Removal via Remote Delamination and Magnetization of Clot Debris**

Qinglong Wang^1,2^, Ben Wang^1^*, Kai Fung Chan^3,4^, Xin Song ^2^, Qianqian Wang^5^, Fengtong Ji^2^, Lin Su^2^, Bonaventure Yiu Ming Ip^6^, Ho Ko^6^, Philip Wai Yan Chiu^3,4,7^, Thomas Wai Hong Leung^6^, Li Zhang^2,3,4,7,8^

^1^College of Chemistry and Environmental Engineering, Shenzhen University, Shenzhen, China.

^2^Department of Mechanical and Automation Engineering, The Chinese University of Hong Kong (CUHK), Shatin, N.T., Hong Kong, China.

^3^Chow Yuk Ho Technology Center for Innovative Medicine, CUHK, Shatin, N.T., Hong Kong, China.

^4^Multi-Scale Medical Robotics Center, Hong Kong Science Park, Shatin N.T., Hong Kong SAR, China.

^5^Jiangsu Key Laboratory for Design and Manufacture of Micro-Nano Biomedical Instruments, School of Mechanical Engineering, Southeast University, Nanjing, China

^6^Division of Neurology, Department of Medicine and Therapeutics, CUHK, Shatin, N.T., Hong Kong, China.

^7^Department of Surgery, CUHK, Shatin, N.T., Hong Kong, China.

^8^CUHK T Stone Robotics Institute, CUHK, Shatin, N.T., Hong Kong, China.

*Corresponding author. Email: B.W.: benwang@szu.edu.cn.

The PDF file includes:

Figure S1. TEM image of pure Fe_3_O_4_ nanoparticles.

Figure S2. XRD patterns of Fe_3_O_4_@SiO_2_ and Fe_3_O_4_ nanoparticles.

Figure S3. Zeta potential and DLS test.

Figure S4. Scanning electron microscope (SEM) image, energy-dispersive X-ray spectroscopy (EDS) image, and element spectrum of Fe_3_O_4_ and Fe_3_O_4_@SiO_2_ NNPs.

Figure S5. Side view of thrombolysis and detachment process for mode 1.

Figure S6. Sequential thrombolysis and detachment of the blood clot from one side.

Figure S7. Investigation of the tPA-microswarm state in different cases.

Figure S8. The magnetic field generated by a 25 mm-diameter NdFeB permanent magnet.

Figure S9. Results of in vitro thrombolysis experiment using an electromagnetic system.

Figure S10. Controlled thrombolysis in a uniform manner and a one-side manner.

Figure S11. Micro-CT scanning of detached blood clot debris.

Figure S12. Mean particle radius of various samples.

Figure S13. The influence of the blood clot mass and the applied magnetic field strength on the movability of clot debris.

Figure S14. The influence of the NNPs content on the movability of clot debris.

Figure S15. Reducing the blood flow to allow retrieval of tPA-microswarm and clot debris by the catheter balloon.

Figure S16. *In vivo* safety analysis of the tPA-labeled microswarms intravenous injection.

Other Supplementary Material for this manuscript includes the following:

Video S1. Thrombolysis and detachment process of two different modes.

Video S2. Thrombolysis process using Fe3O4 nanoparticles.

Video S3. Detachment process accelerated by torsion.

Video S4. Thrombolysis by tPA-microswarm with an electromagnetic system.

Video S5. Typical locomotion of detached magnetic blood clot debris in the whole blood.

Video S6. Locomotion ability of the detached blood clots with random shapes.

Video S7. 3D structure of the detached blood clot debris under micro-CT scanning

Video S8. Fast detachment and remote retrieval of the blood clot in the phantom.


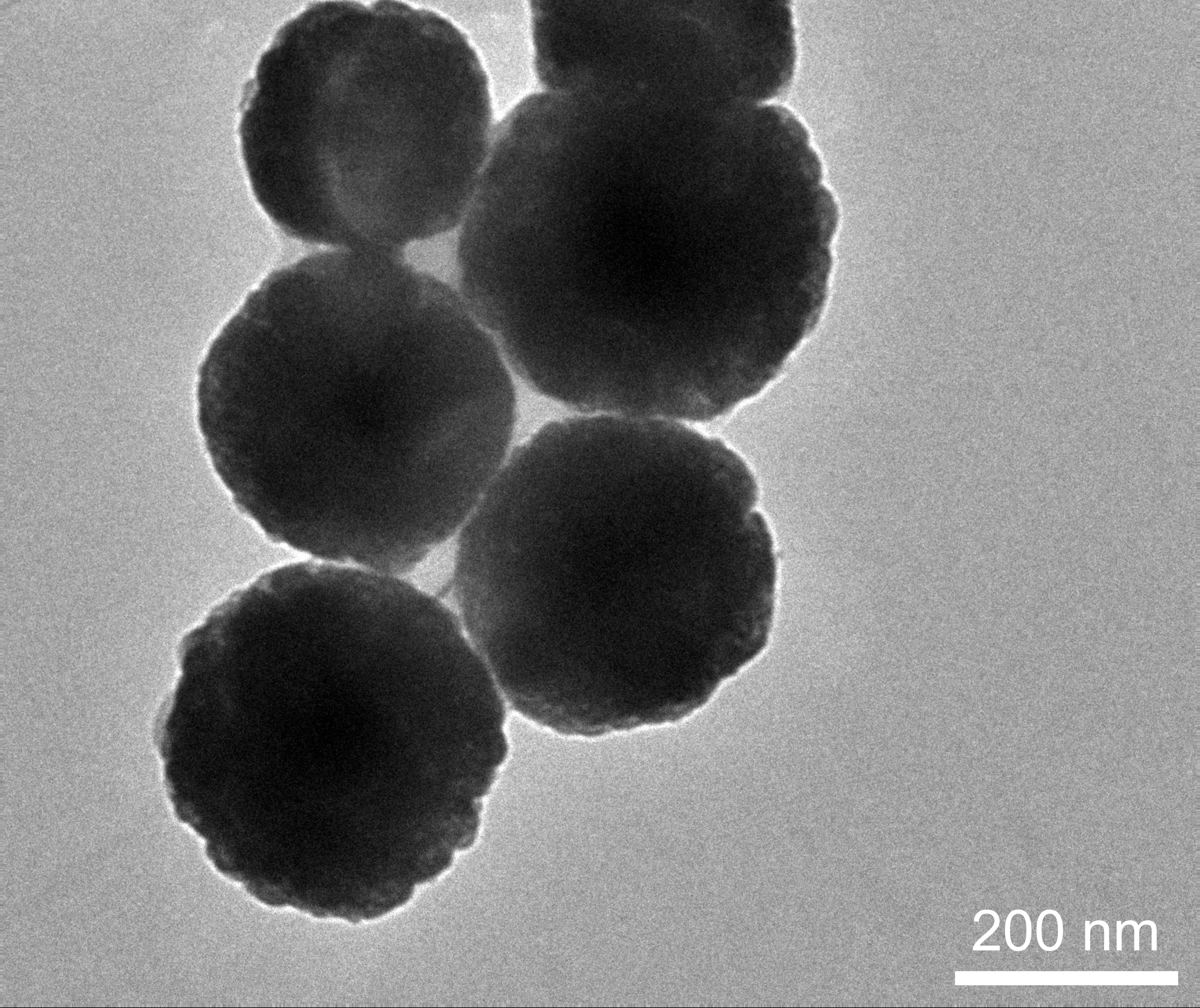
Figure S1. TEM image of pure Fe_3_O_4_ nanoparticles.


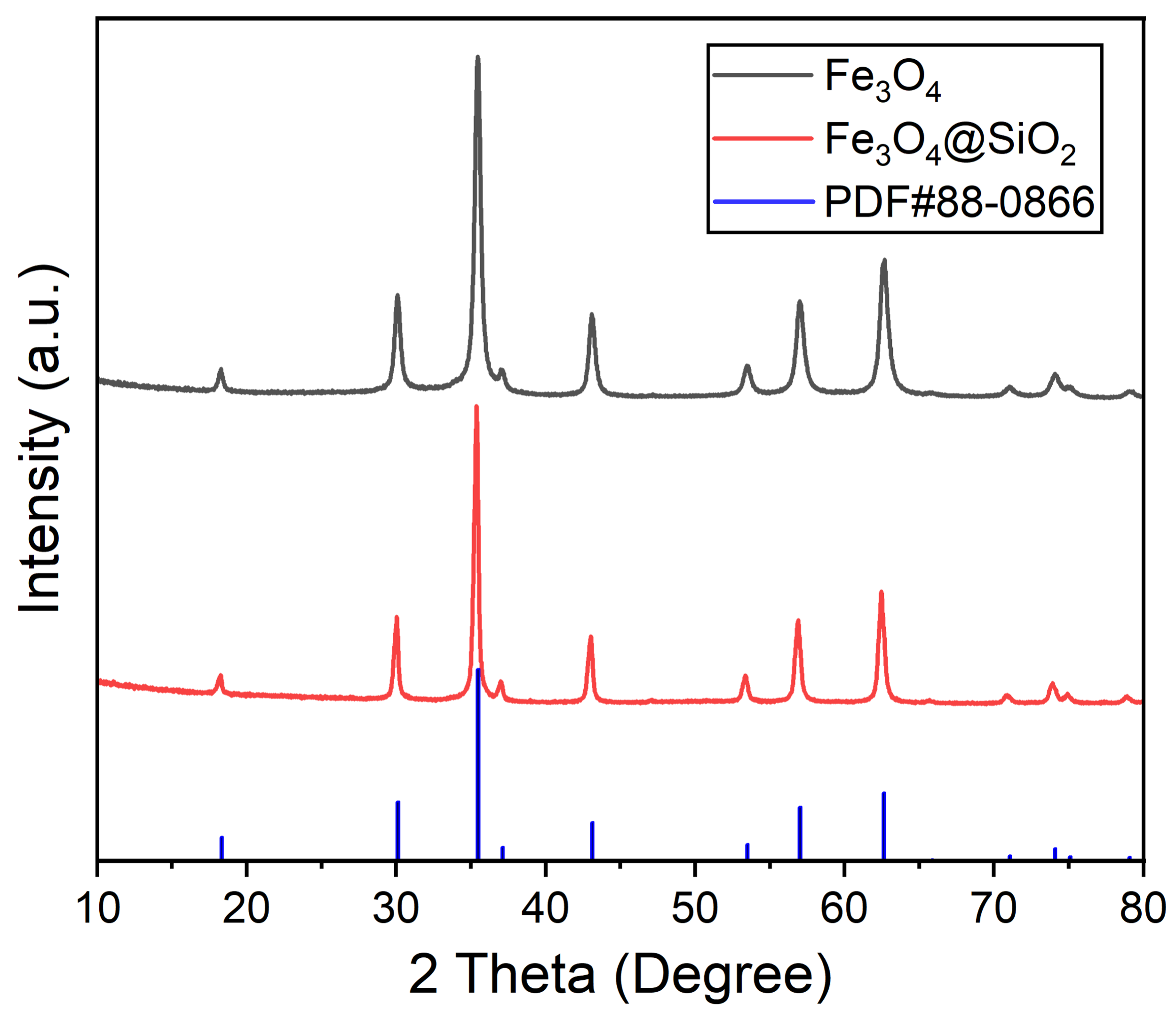
Figure S2. XRD patterns of Fe_3_O_4_@SiO_2_ and Fe_3_O_4_ nanoparticles.


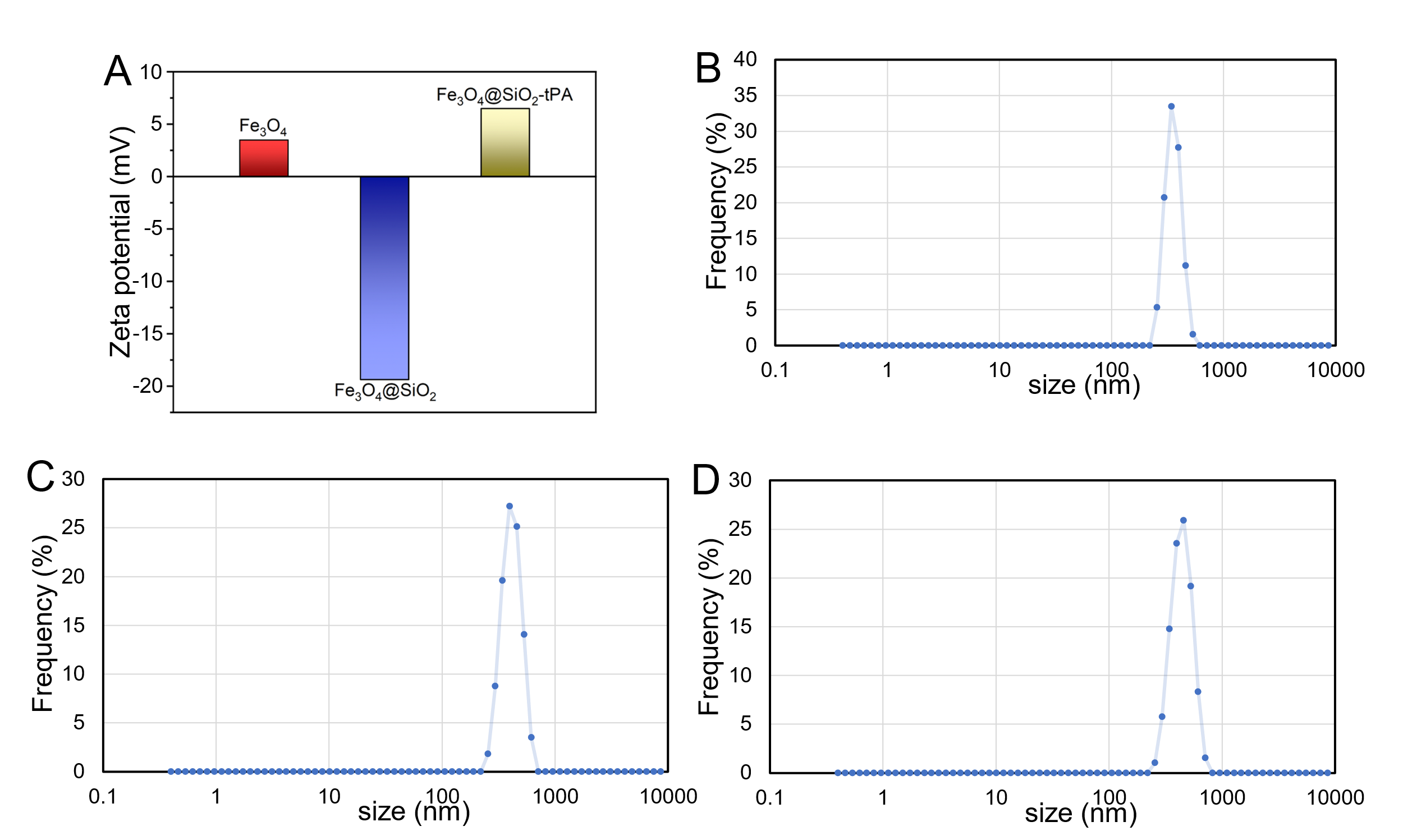
Figure S3. Zeta potential and DLS test. (A) Zeta potentials of Fe_3_O_4_, Fe_3_O_4_@SiO_2_, and Fe_3_O_4_@SiO_2_-tPA NNPs in water. (B-D) Dynamic light scattering (DLS) of Fe_3_O_4_, Fe_3_O_4_@SiO_2_, and Fe_3_O_4_@SiO_2_-tPA NNPs in water, respectively.


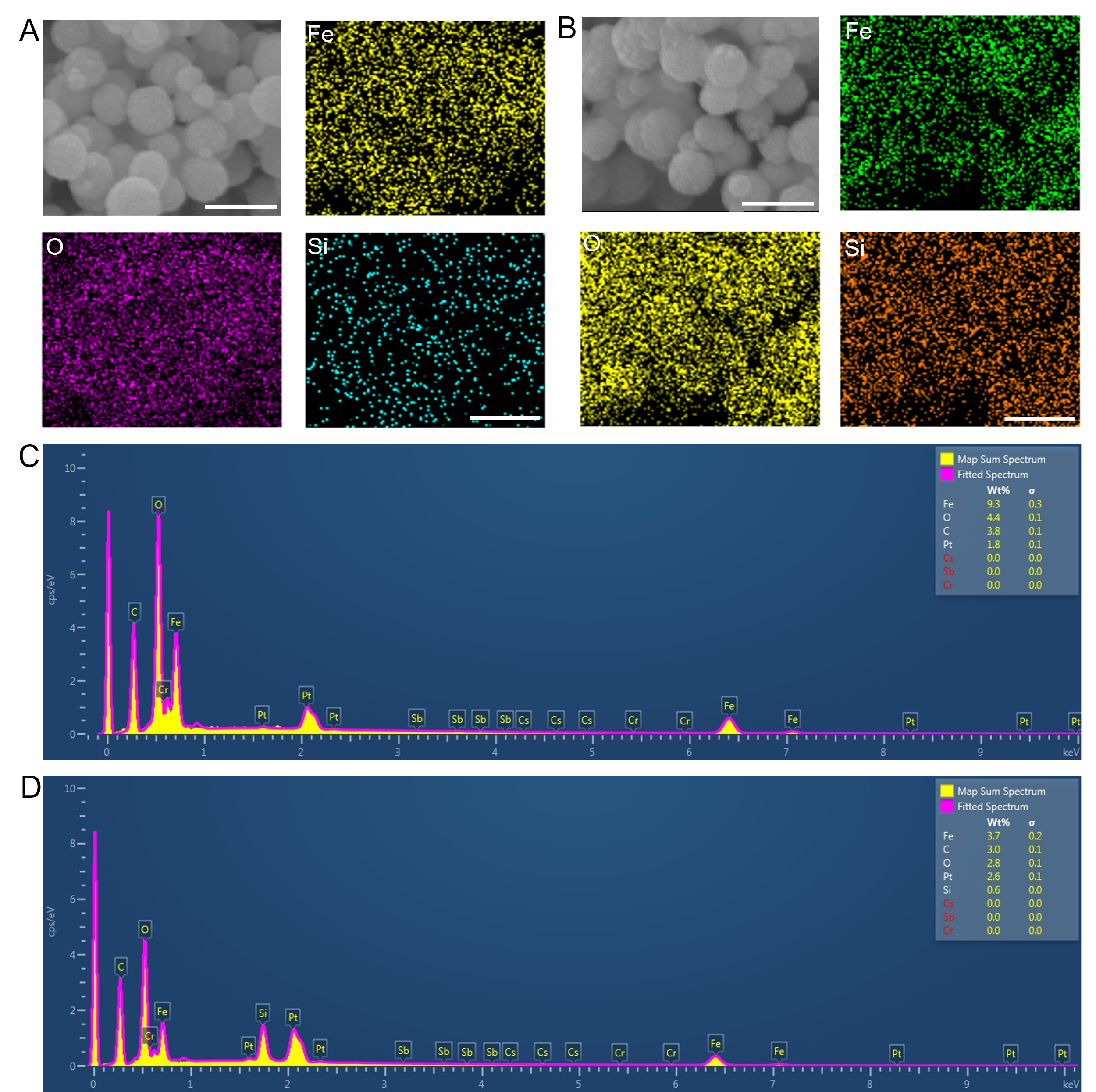
**Figure S4. Scanning electron microscope (SEM) image, energy-dispersive X-ray spectroscopy (EDS) image, and element spectrum of Fe_3_O_4_ and Fe_3_O_4_@SiO_2_ NNPs.** (**A-B**) SEM and EDS images of Fe_3_O_4_, and Fe_3_O_4_@SiO_2_ NNPs, respectively. All scale bars are 500 nm. (**C-D**) Element spectrums of Fe_3_O_4_, Fe_3_O_4_@SiO_2_ NNPs, respectively.


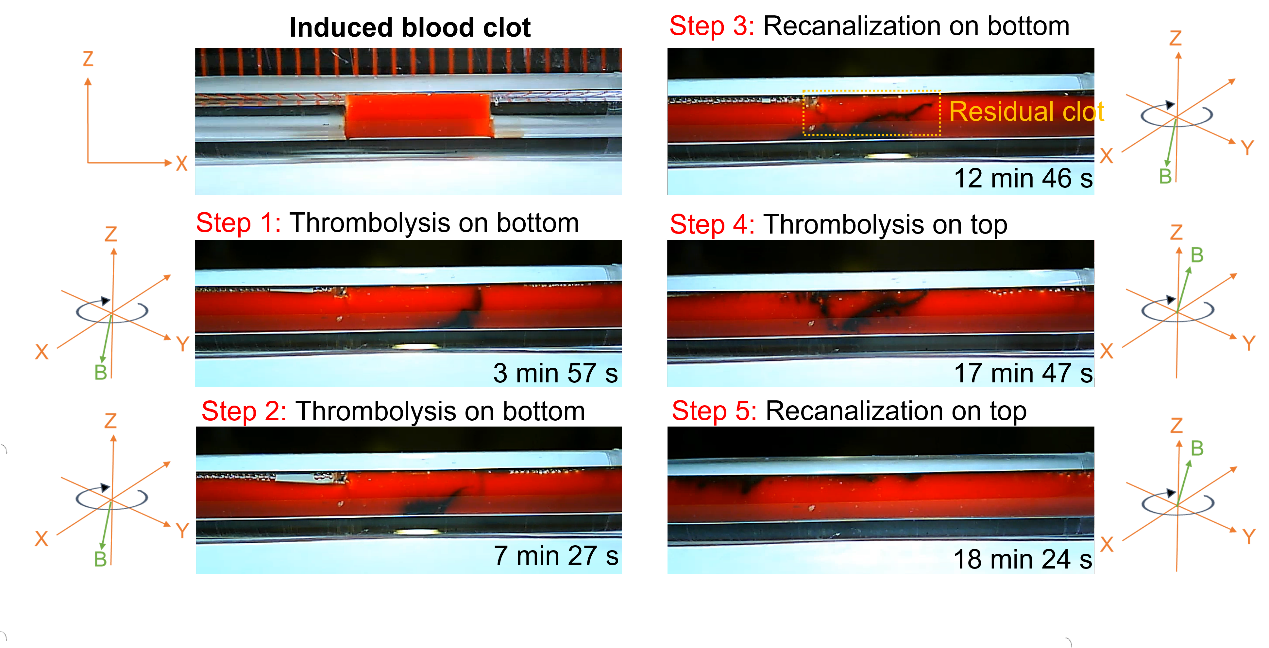


Figure S5. Side view of thrombolysis and detachment process for mode 1. Step 1, thrombolysis in the vessel bottom was realized by applying the rotating magnetic field generated by a sphere magnet; Step 2, continuous thrombolysis on the vessel bottom; Step 3, fast recanalization on the bottom was achieved; Step 4, the rotating magnetic field was adjusted to attract the tPA-microswarm to the vessel top and lyse the blood clot; Step 5, the blood clot was fully detached from the tube wall. Experimental parameters: magnetic field strength, 64.9 mT; frequency, 3Hz; the dose of tPA-microswarm, 1 mg; blood, whole blood.


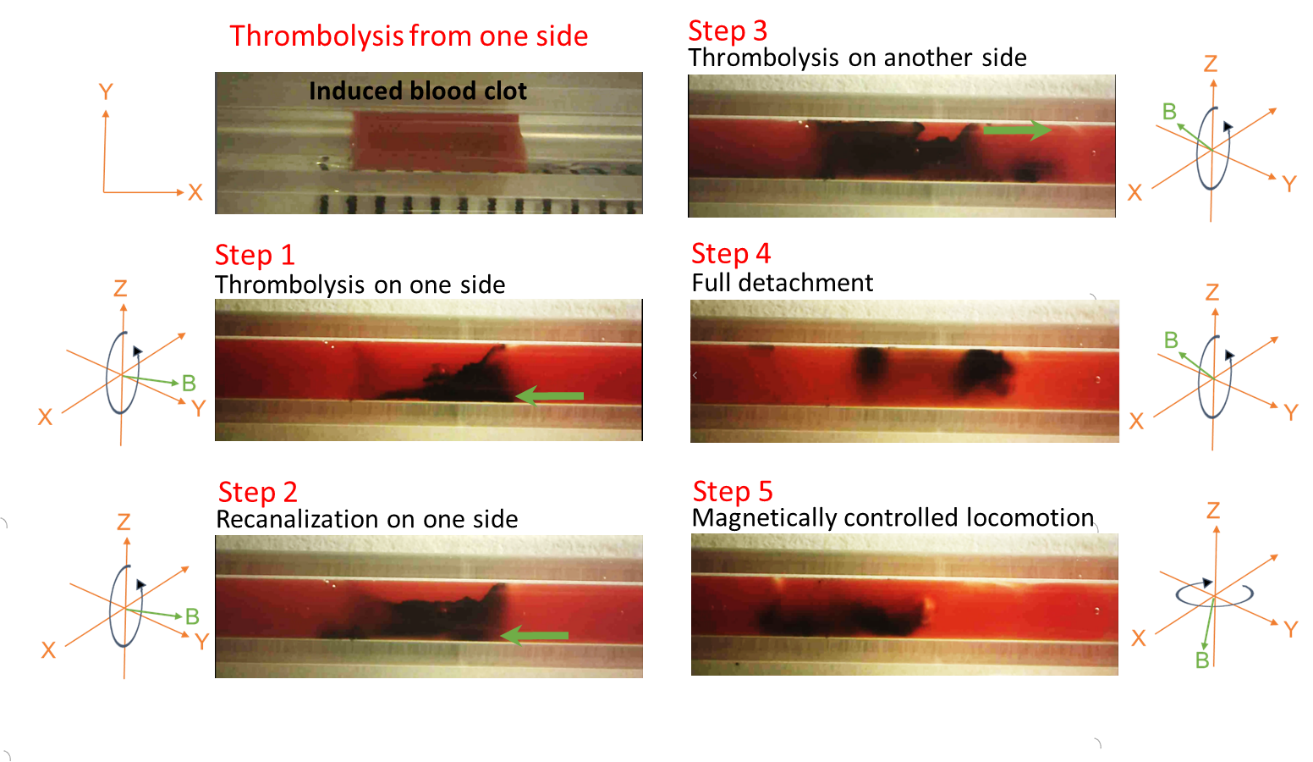


Figure S6. Sequential thrombolysis and detachment of the blood clot from one side. Step 1, thrombolysis from one side of the vessel was realized by applying the rotating magnetic field generated by a sphere magnet; Step 2, fast recanalization on one side of the vessel was achieved; Step 3, The rotating magnetic field was adjusted to attract the tPA-microswarm to another side of the vessel and further lysed the blood clot; Step 4, The blood clot was fully detached from the tube wall; Step 5, tPA-microswarm mixed with magnetic debris was able to locomote for the further retrieval process. Experimental parameters: magnetic field strength, 64.9 mT; frequency, 3Hz; the dose of tPA-microswarm, 1 mg; blood, whole blood.


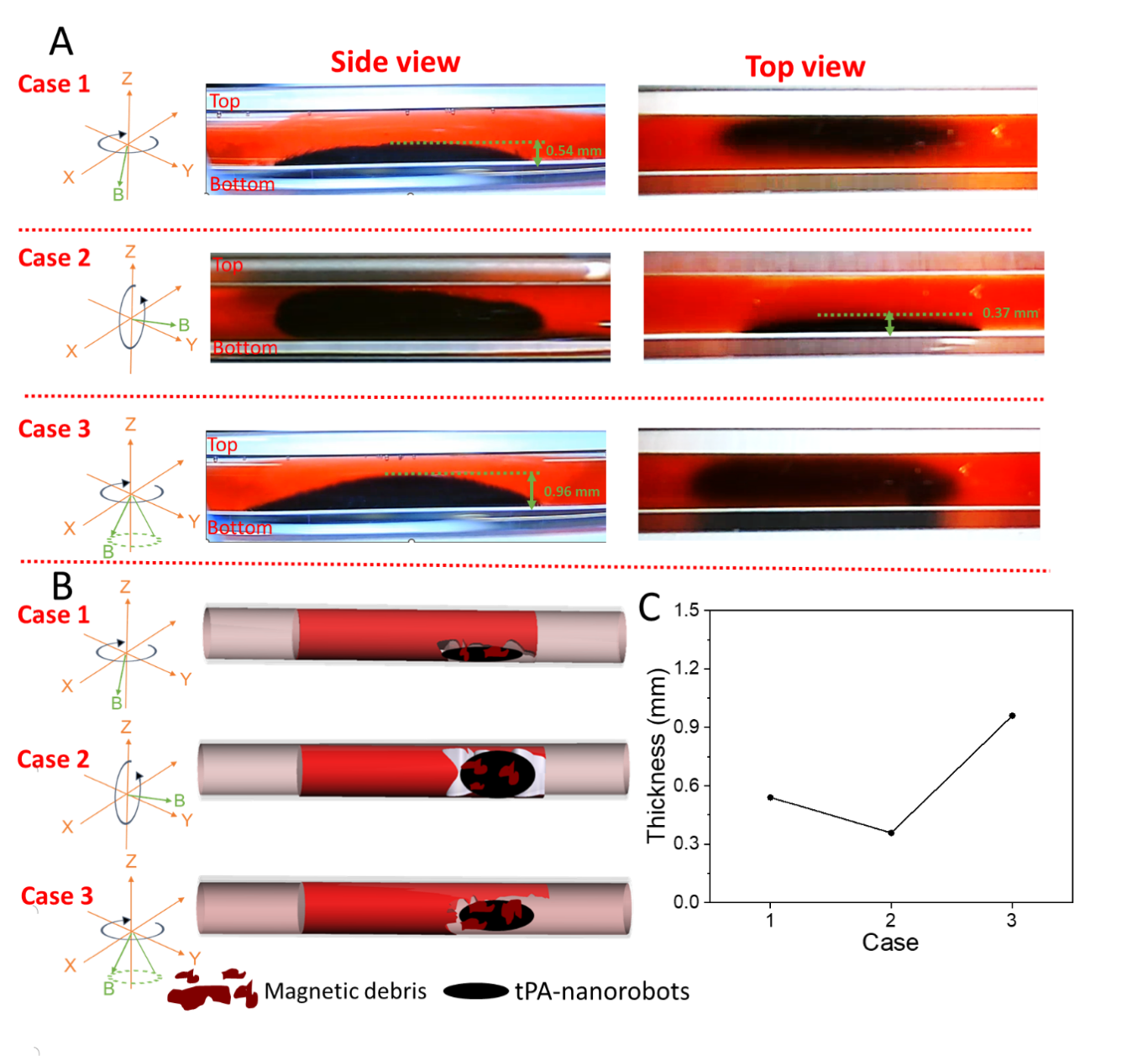


Figure S7. Investigation of the tPA-microswarm state in different cases. (A) The top view and side view show the swarm state in the tube with a diameter of 1.5 mm. Case 1, the rotating magnet was applied from the tube bottom; Case 2, the rotating magnet was applied from one side of the tube; Case 3, the rotating magnet was applied from the tube bottom with a conical rotating magnetic field. (B) Schematic of the tPA-nanorobots lysing the blood clot with three cases. (C) The thickness of tPA-microswarm in different cases.


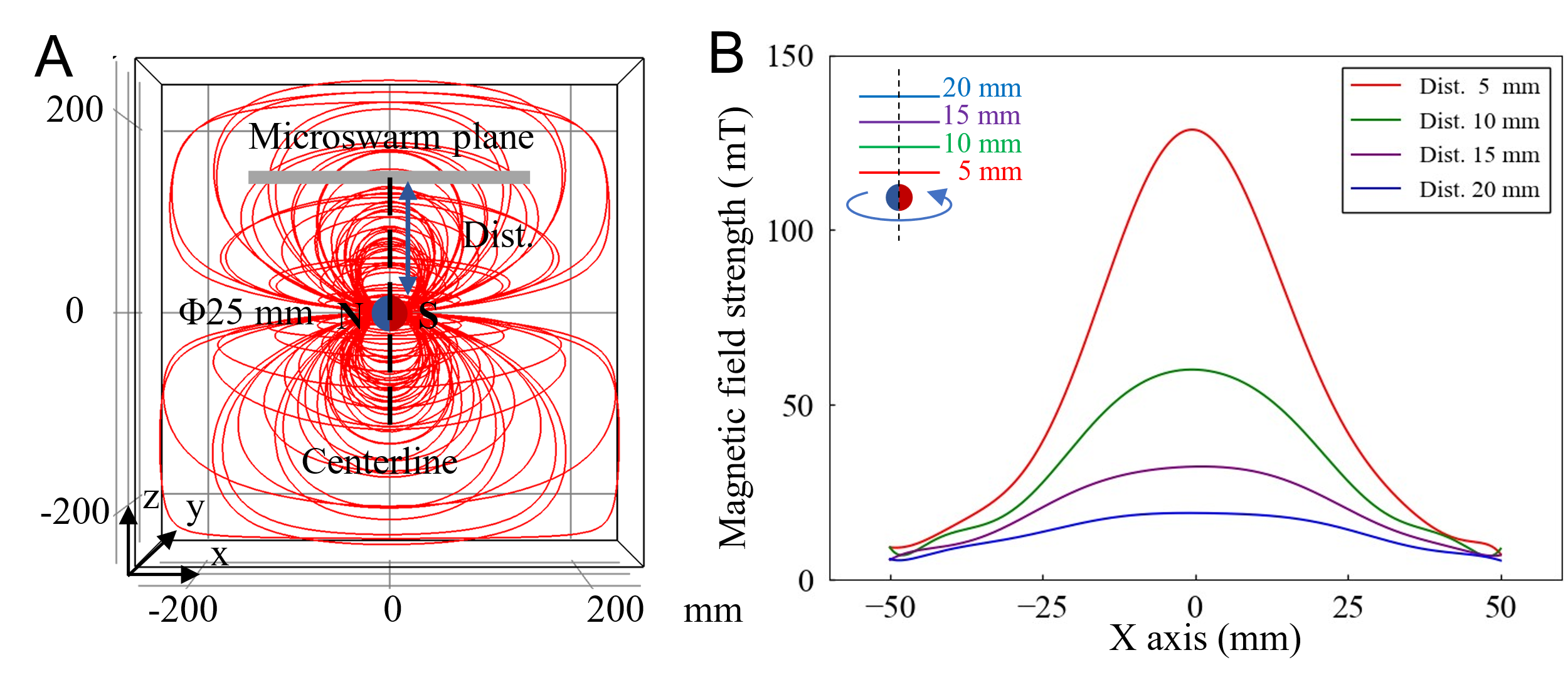
Figure S8. The magnetic field generated by a 25 mm-diameter NdFeB permanent magnet. (A) Magnetic field distribution formed by a 25 mm-diameter sphere magnet. (B) Field strength along the X-axis at a distance (dist.) of 5, 10, 15, and 20 mm to the tip surface of the magnet. Lines denote fitting curves of simulated data.


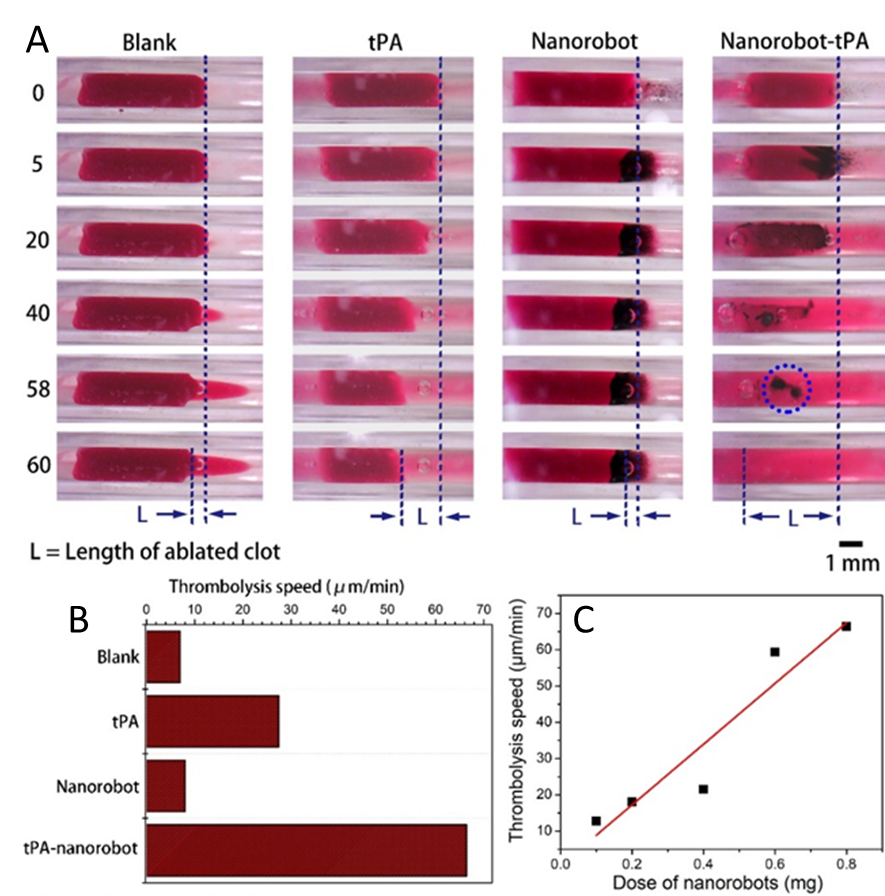


**Figure S9**. **Results of in vitro thrombolysis experiment using an electromagnetic system.** (**A**) Schematic and video clips show the thrombolysis of four experimental groups at different time intervals under 37°C with a designed magnetic field (strength is 9 mT, frequency is 3 Hz, and the direction angle is 90°C). The groups from left to right, in turn, are the saline solution, 50 μL of 2.5 mg/mL tPA, 50 μL of 1 mg/mL nanorobots (NNPs), and 50 μL of 1 mg/mL of tPA-nanorobots (tPA labeled NNPs). The amount of tPA in Group 4 is less than 1% of the amount of tPA in Group 2. (**B**) The bar graph shows the thrombolysis speed of different groups. (**C**) Relationship between the thrombolysis speed and the dose of the tPA-nanorobot.


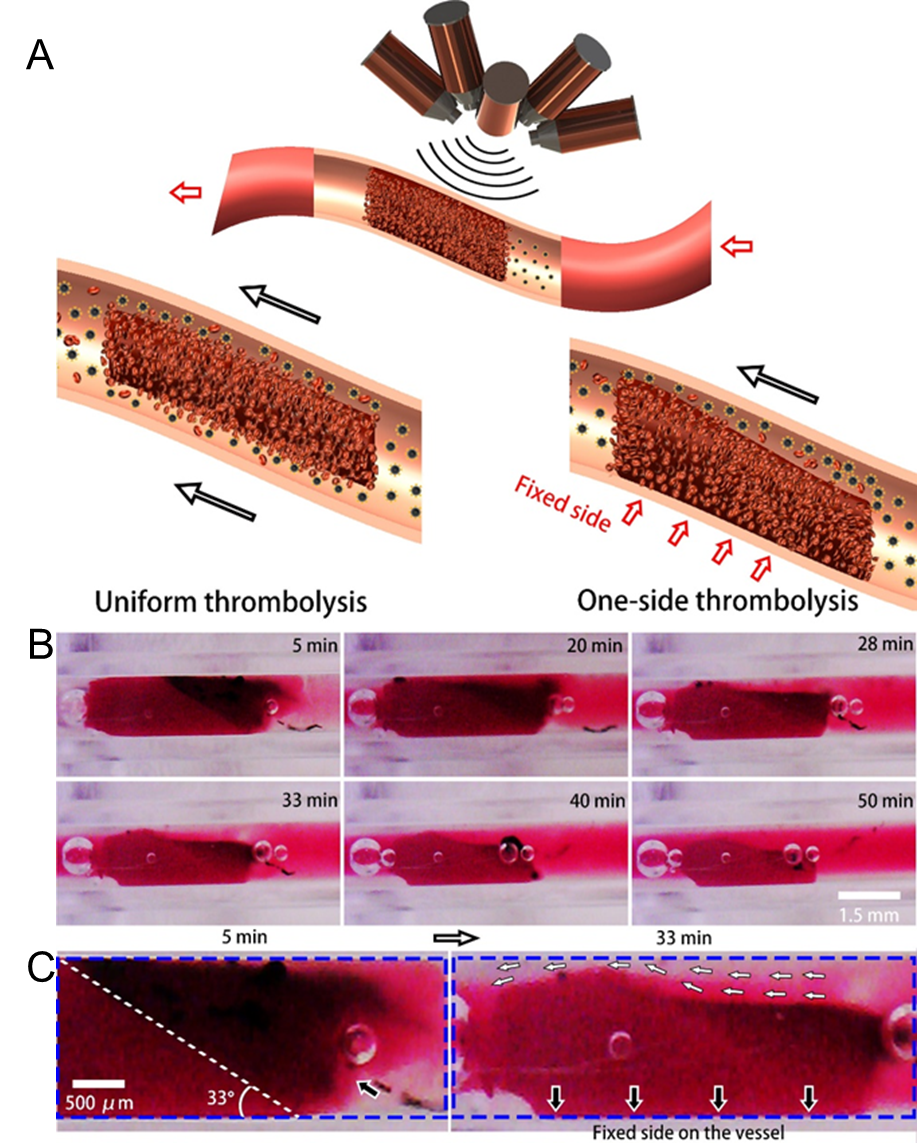
Figure S10. Controlled thrombolysis in a uniform manner and a one-side manner. (A) Schematic illustration of the controlled thrombolysis in a uniform manner and a one-side manner. (B) Successive images show the selective thrombolysis of one side of the blocked vessels. (C) Enlarged views show the thrombolysis at 5 min and 33 min.


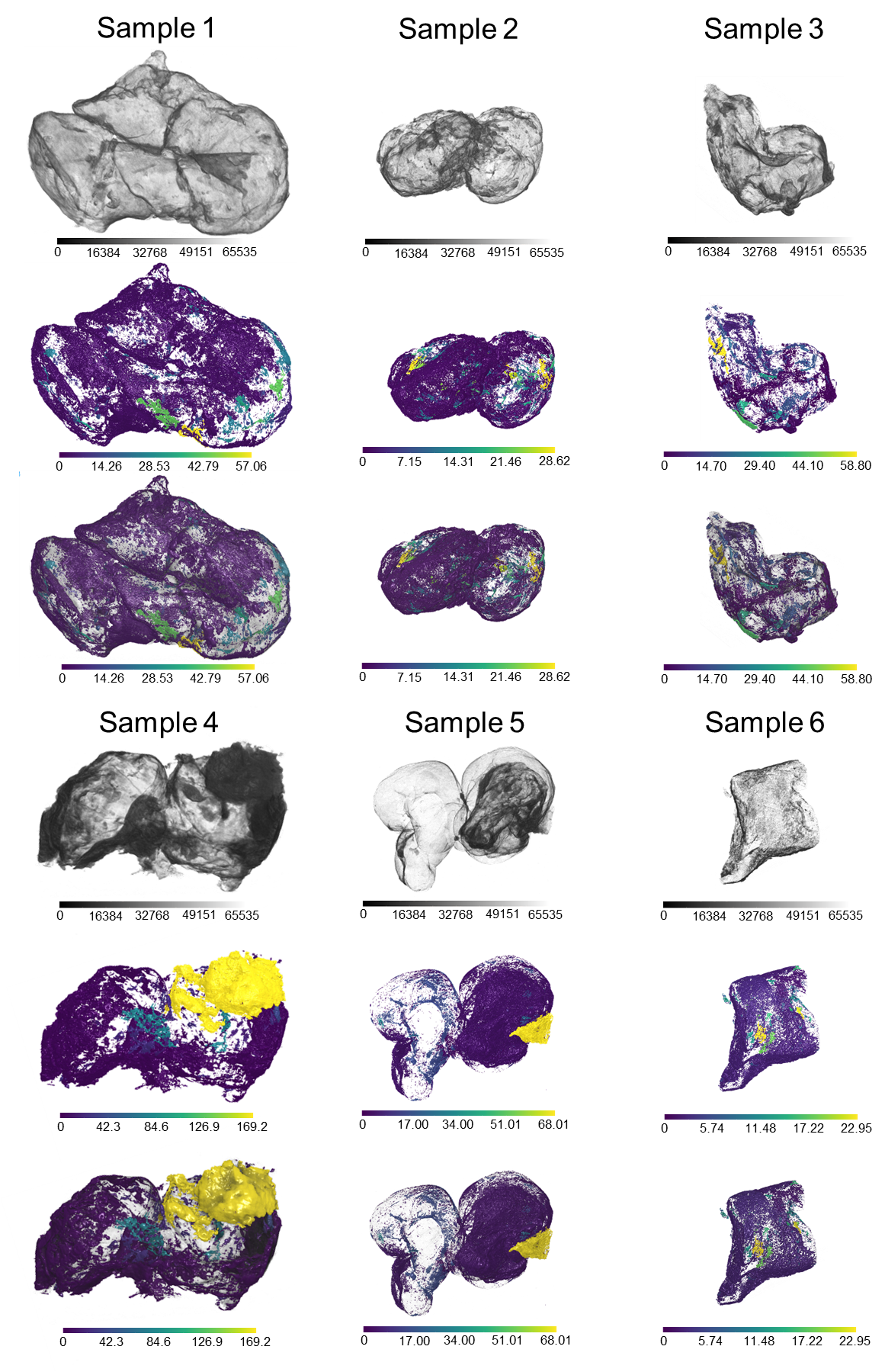
Figure S11. Micro-CT scanning of detached blood clot debris. Various samples with 3D scanning of the blood clot debris, 3D rendering of the blood clot debris after particle segmentation and extraction, and overlap of 3D scanning and rendering of the blood clot debris.

Figure S12. Mean particle radius of various
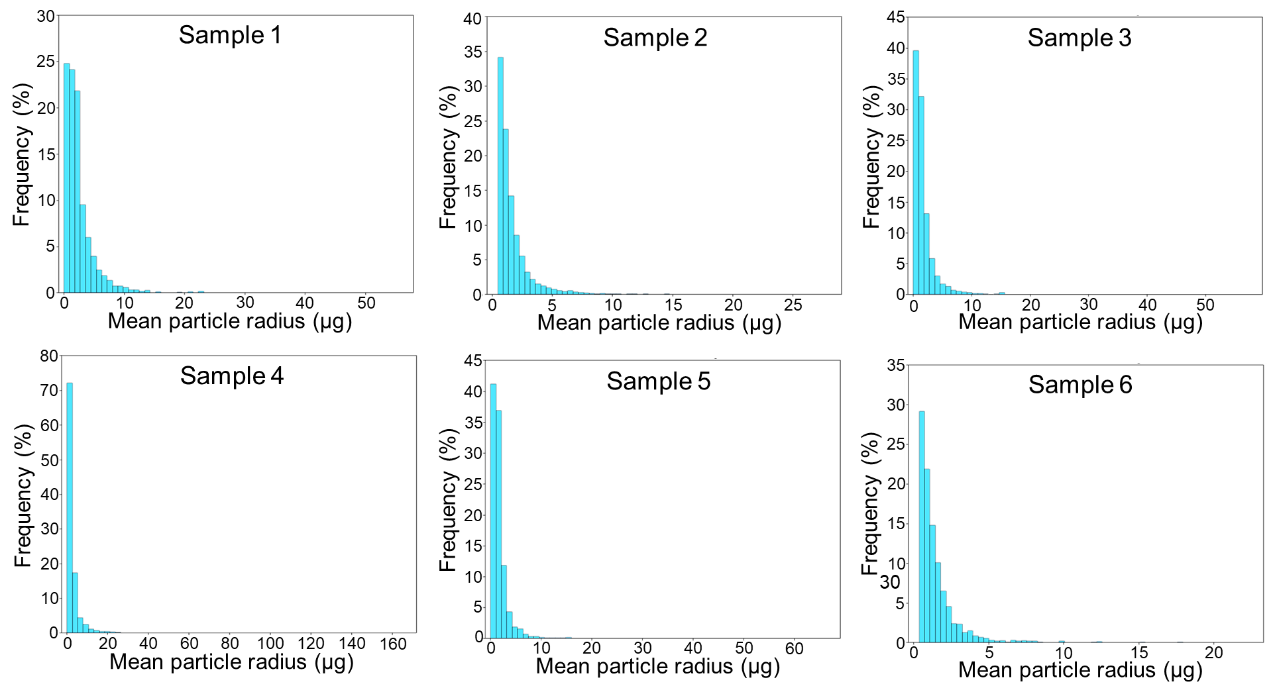
samples.


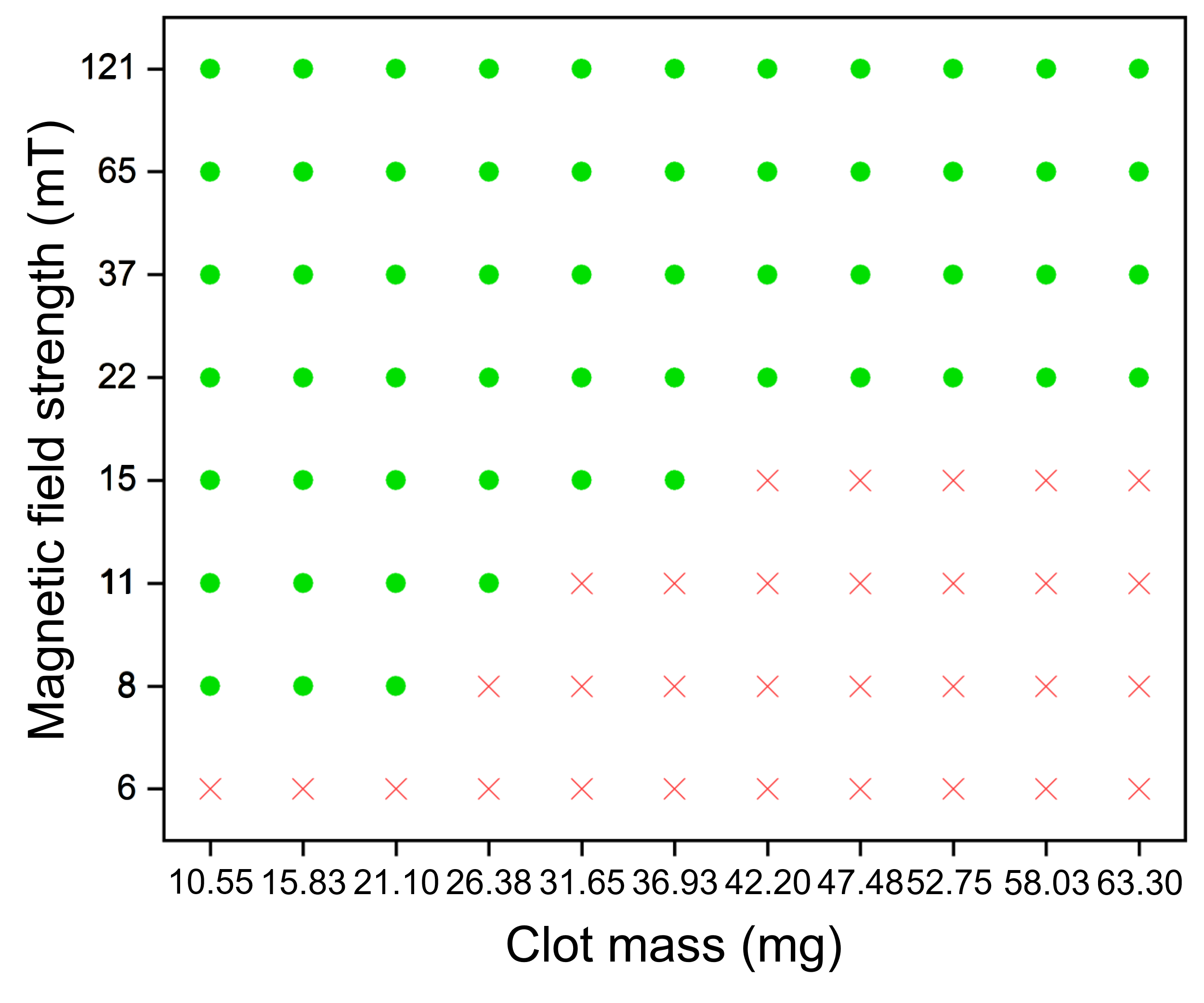
**Figure S13. The influence of the blood clot mass and the applied magnetic field strength on the movability of clot debris.** Phase diagram of the movability of the blood clot debris with various magnetic field strengths and mass contents, where green dots indicate movability, and red crosses indicate non-movability of the debris. The weight of incorporated magnetic NNPs was 1 mg, and the input magnetic field frequency was 4 Hz.


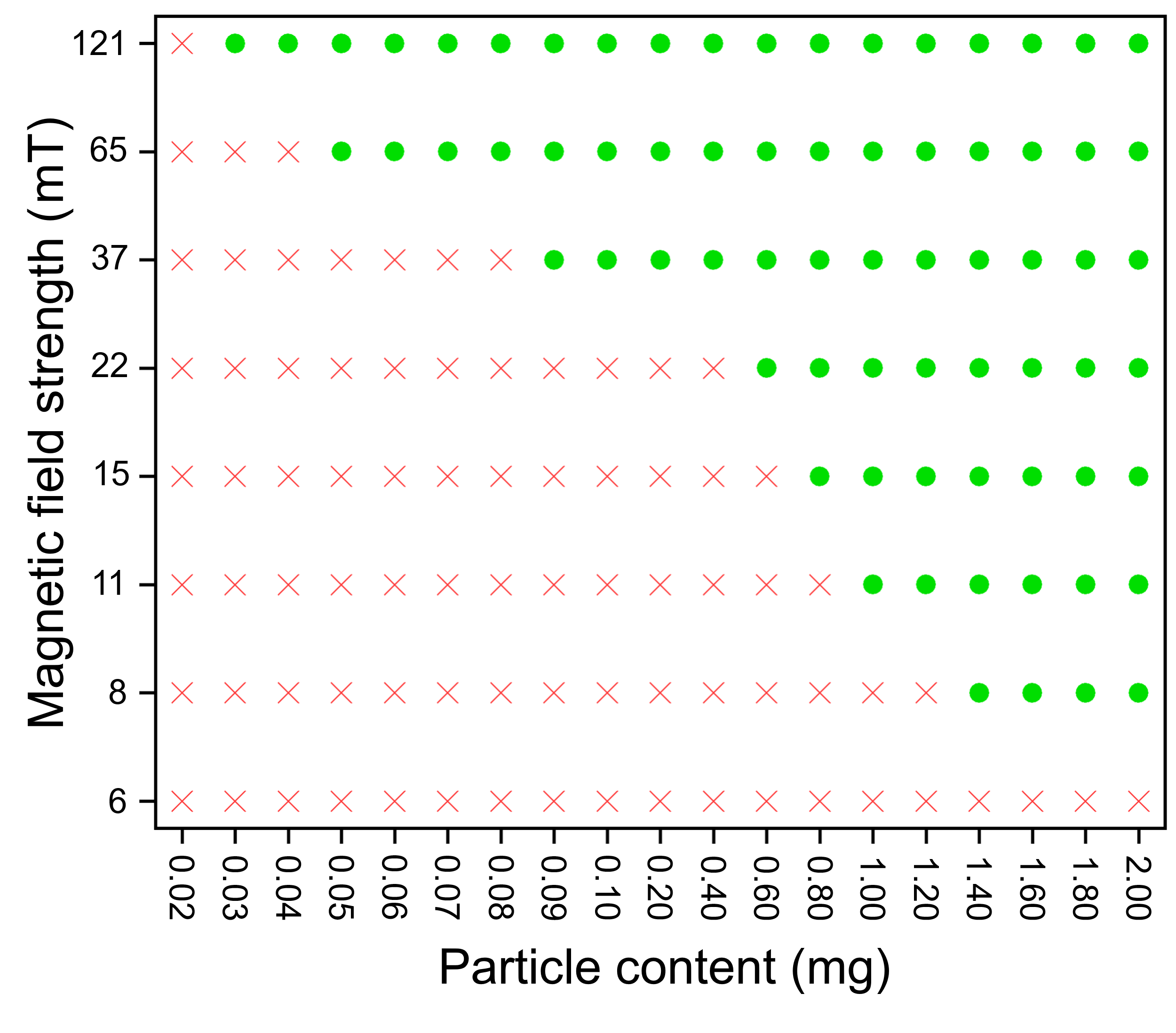


**Figure S14. The influence of the NNPs content on the movability of clot debris.** Phase diagram of the movability of the blood clot debris with various magnetic field strengths and NNPs contents, where green dots indicate movability, and red crosses indicate non-movability of the debris. The weight of the incubated clot was 21.10 mg, and the input magnetic field frequency was 4 Hz.


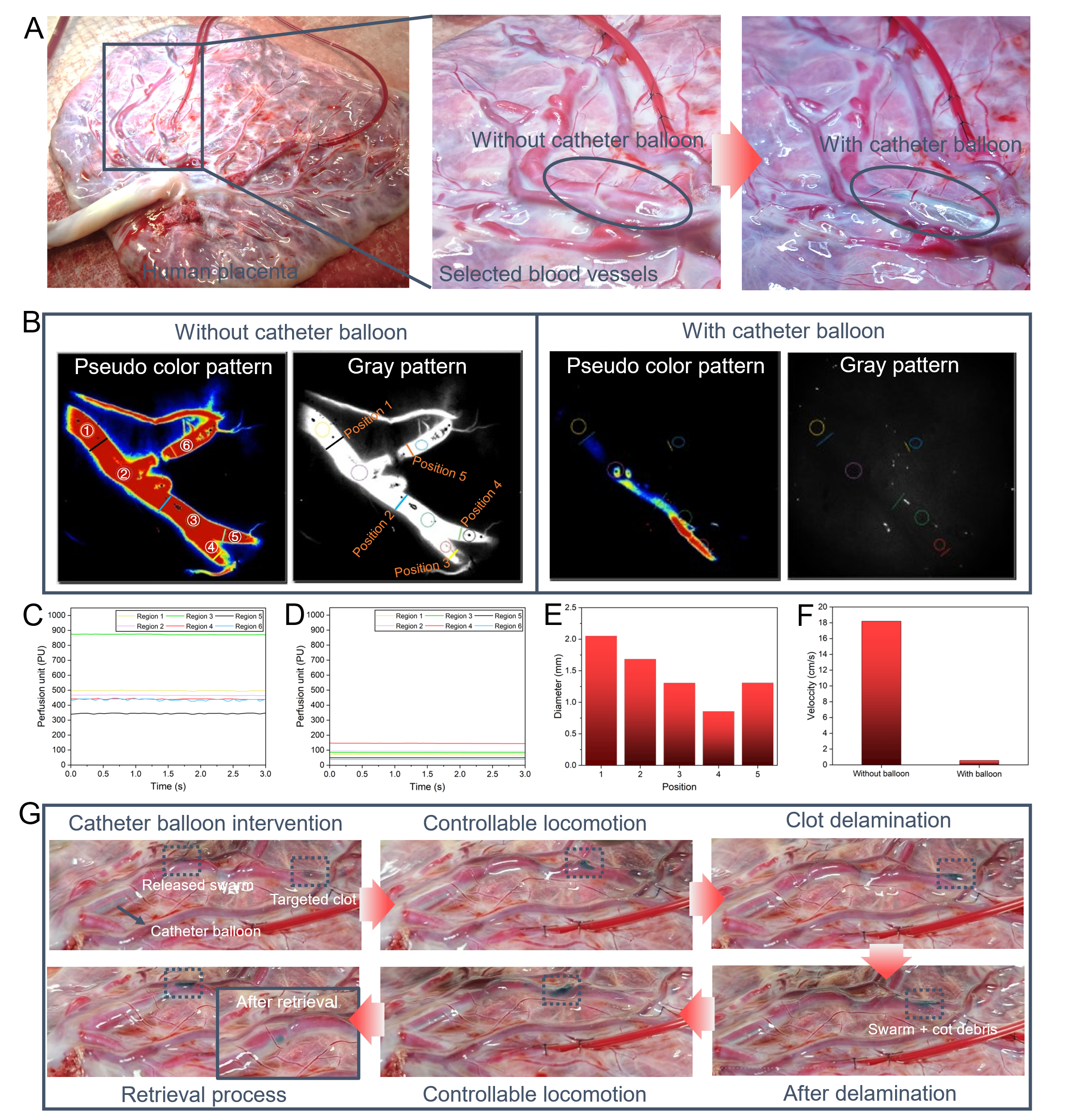
**Figure S15. Reducing** **the blood flow to allow retrieval of tPA-microswarm and clot debris by the catheter balloon.** (**A**) The left photo shows the vascular distribution of the human placenta, the middle photo shows the selected blood vessels used as models, and the right photo shows the blood vessel with catheter balloon intervention. (**B**) Laser speckle contrast imaging (LSCI) of the model blood vessel with or without catheter balloon intervention. Regions ①-⑥ are typical areas for indicating the perfusion unit (PU) change. Positions 1-5 are used for measuring the diameter of the blood vessels. (**C-D**) PU change of various regions with or without catheter balloon intervention, respectively. (**E**) The average diameters of the blood vessel at different positions. (**F**) The mean blood flow velocity at position 3 with or without catheter balloon intervention. (**G**) The demonstration process in the human placenta using the catheter balloon for reducing the flow velocity.


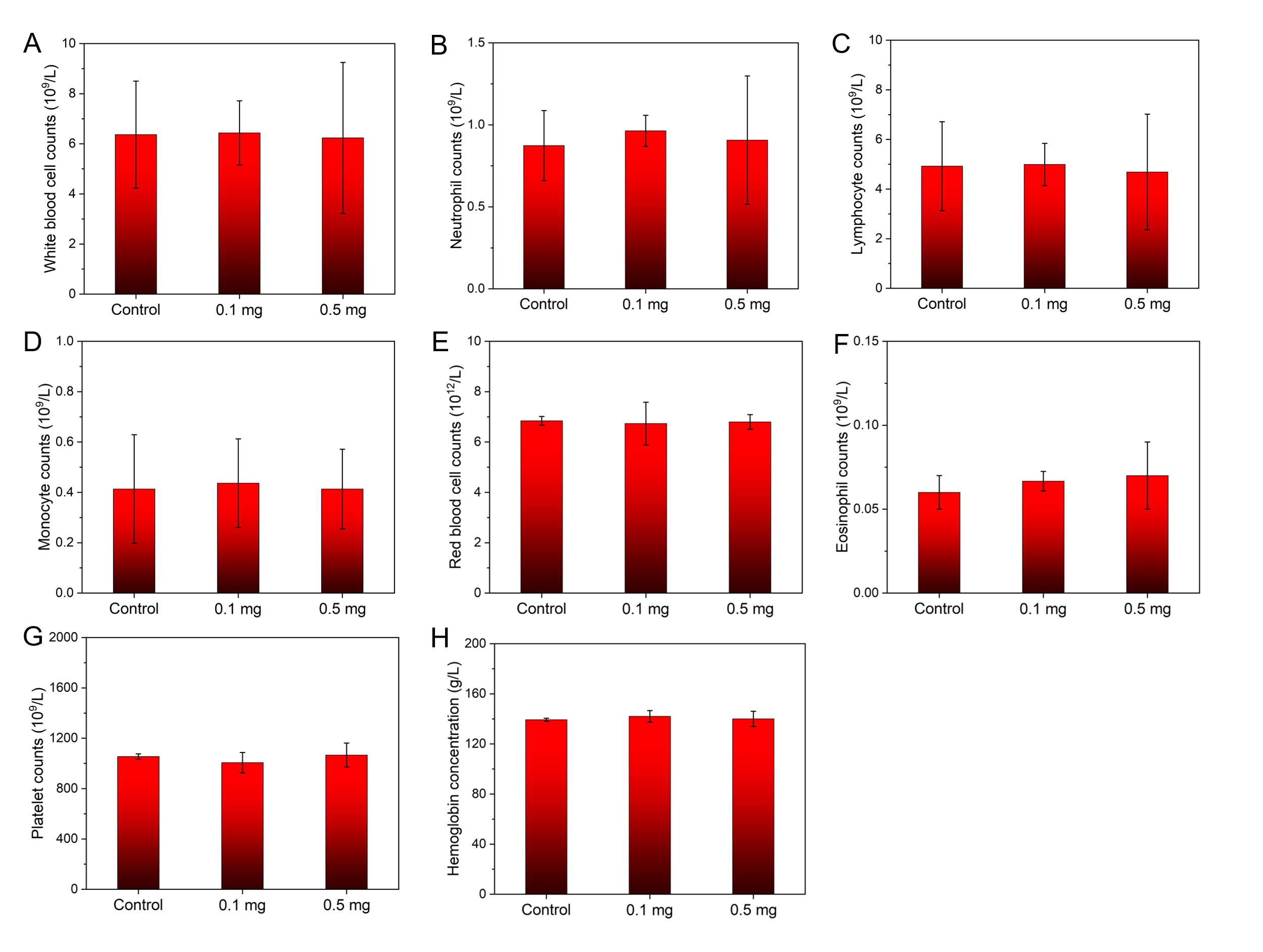
**Figure S16. *In vivo* safety analysis of the tPA-labeled microswarms intravenous injection.** （A-H）Comprehensive blood analysis panel of rats injected with PBS (control), and rats injected with tPA-labeled NNPs (0.1 mg, and 0.5 mg) (n = 3). Blood samples were collected on day 7.
